# Captive breeding of specialty animals represents an overlooked yet critical reservoir for spreading antibiotic resistance genes

**DOI:** 10.1093/ismejo/wrag009

**Published:** 2026-01-28

**Authors:** Jiao Xi, Hanxiang Tao, Ziyi Zhang, Biyao Lian, Wei Sun, Yang Zhang, Shuhai Bu, Xiaojun Yang, Xun Qian

**Affiliations:** College of Natural Resources and Environment, Northwest A&F University, 3 Taicheng Rd, Yangling, Shaanxi 712100, China; Interdisciplinary Research Center for Soil Microbial Ecology and Land Sustainable Productivity in Dry Areas, Northwest A&F University, 3 Taicheng Rd, Yangling, Shaanxi 712100, China; College of Natural Resources and Environment, Northwest A&F University, 3 Taicheng Rd, Yangling, Shaanxi 712100, China; Interdisciplinary Research Center for Soil Microbial Ecology and Land Sustainable Productivity in Dry Areas, Northwest A&F University, 3 Taicheng Rd, Yangling, Shaanxi 712100, China; College of Natural Resources and Environment, Northwest A&F University, 3 Taicheng Rd, Yangling, Shaanxi 712100, China; Interdisciplinary Research Center for Soil Microbial Ecology and Land Sustainable Productivity in Dry Areas, Northwest A&F University, 3 Taicheng Rd, Yangling, Shaanxi 712100, China; College of Natural Resources and Environment, Northwest A&F University, 3 Taicheng Rd, Yangling, Shaanxi 712100, China; Interdisciplinary Research Center for Soil Microbial Ecology and Land Sustainable Productivity in Dry Areas, Northwest A&F University, 3 Taicheng Rd, Yangling, Shaanxi 712100, China; College of Natural Resources and Environment, Northwest A&F University, 3 Taicheng Rd, Yangling, Shaanxi 712100, China; Interdisciplinary Research Center for Soil Microbial Ecology and Land Sustainable Productivity in Dry Areas, Northwest A&F University, 3 Taicheng Rd, Yangling, Shaanxi 712100, China; College of Natural Resources and Environment, Northwest A&F University, 3 Taicheng Rd, Yangling, Shaanxi 712100, China; Interdisciplinary Research Center for Soil Microbial Ecology and Land Sustainable Productivity in Dry Areas, Northwest A&F University, 3 Taicheng Rd, Yangling, Shaanxi 712100, China; College of Life Sciences, Northwest A&F University, 3 Taicheng Rd, Yangling, Shaanxi 712100, China; College of Animal Science and Technology, Northwest A&F University, 3 Taicheng Rd, Yangling, Shaanxi 712100, China; College of Natural Resources and Environment, Northwest A&F University, 3 Taicheng Rd, Yangling, Shaanxi 712100, China; Interdisciplinary Research Center for Soil Microbial Ecology and Land Sustainable Productivity in Dry Areas, Northwest A&F University, 3 Taicheng Rd, Yangling, Shaanxi 712100, China

**Keywords:** specialty animal breeding, captivity, ARG transmission, resistome, musk deer

## Abstract

Driven by wildlife conservation and economic demands, captive breeding has expanded globally, intensifying wildlife–human interactions. In specialty animal breeding, particularly for species with short domestication histories and underdeveloped breeding protocols, clinically important antibiotics are commonly misused, posing potential ecological and health risks that remain largely unexplored. We collected fecal samples from three groups of musk deer (*Moschus berezovskii*): those exposed to clinically important antibiotics, those not exposed for six months, and wild musk deer, and analyzed their microbiomes and resistomes using metagenomic and culture-based methods. We found that captivity significantly expanded and reshaped the fecal resistome of musk deer. The antibiotic-exposed musk deer harbored a significantly higher diversity and abundance of antibiotic resistance genes (ARGs) compared to those non-exposed to antibiotics and wild deer. We observed a higher abundance of clinically important ARGs within *Enterobacteriaceae* in fecal samples of captive musk deer. This observation was further supported by the antibiotic susceptibility profiles of 124 *Escherichia coli* strains isolated from antibiotic-exposed musk deer. Seven identical mobile genetic element-associated ARGs were detected in distinct bacterial hosts across fecal samples from musk deer and farm workers, indicating potential conjugative transfer between the two groups. Our results suggest that captive breeding of specialty animals is an overlooked but significant reservoir for disseminating clinically important ARGs, and underscore the transmission risk at the animal–human interface.

## Introduction

Antibiotic resistance poses a severe threat to global public health, claiming 4.95 million lives annually and continuing to escalate [1–3]. The expansion of urbanization and agriculture development intensifies contacts between wildlife, livestock, and humans, elevating risks of pathogen and antibiotic resistance genes (ARGs) transmission across species boundaries and threatening wildlife and human health. Many pandemics in human history have been related to wild animals. About 75% of human pathogens originate from wild animals [4], such as plague and several recent viral pandemics [5]. Wildlife behaviors, such as foraging and migration, facilitate the spread of microbiomes and resistomes across extensive geographic ranges and, ultimately, into human environments [6]. For instance, migratory birds have been implicated in transferring ARGs into human habitats during their journeys [7, 8]. Concurrently, human activities, particularly domestication and captivity, significantly reshape wildlife microbiomes [6]. When domesticated animals are released or escape into the wild and interact with their wild counterparts, the microbiomes they acquire in captivity may compromise wild populations' health and disrupt ecological balance [9, 10].

The musk deer (*Moschus berezovskii*, belonging to *Moschidae*) is a mammal primarily inhabiting mid- to high-altitude forests across Asia. It produces musk—a highly valued traditional Chinese medicine and spice—for which it has been heavily hunted, leading to a severe decline in wild populations and bringing the species to the brink of extinction [11]. In response to this population decline, intensive captive breeding programs have rapidly expanded across Asian countries over the past decade. Due to their short farming history and incomplete breeding practices, musk deer are susceptible to diarrhea, pneumonia, and suppurative diseases [12]. Consequently, clinically important antibiotics such as beta-lactams, fluoroquinolones, and aminoglycosides, are frequently misused in captive musk deer [13, 14], primarily through prophylactic use and empirical treatment of diarrhea. In fact, this represents a common issue in the captive breeding of specialty animals, such as red foxes [15, 16]. Despite the global efforts to limit antibiotic usage in livestock breeding, misusing clinically important antibiotics in specialty animal breeding may serve as an overlooked yet significant pathway for enriching and disseminating clinical ARGs among wildlife and humans. The transmission of ARG-carrying bacteria from farm animals to workers and interns has been reported [17]. The potential risk of ARG transmission from specialty animal breeding and its impact on bacteria in the surrounding environment have not yet been evaluated.

We collected fecal samples from wild and captive musk deer (recently exposed versus not exposed to antibiotics), and feces from farm workers and the local villagers. We analyzed the microbial composition, ARGs and their potential for horizontal transfer using integrated metagenomic sequencing and culture-based methods. This study tests three hypotheses: (i) Captivity alters the fecal resistome of wild musk deer, increasing ARG abundance and diversity; (ii) Misuse of clinically important antibiotics in musk deer farming reshapes the fecal resistome and elevates the risk of ARG dissemination; (iii) ARGs from the fecal microbiomes of captive musk deer may spread to farm workers. Our findings provide insights into the transition in resistome profiles induced by wildlife farming and the health risks mediated by specialty animal breeding. We also highlight the urgency of strict regulation of antibiotic use in specialty animal breeding.

## Materials and Methods

### Sample collection

Fecal samples from musk deer were collected from a large-scale captive breeding farm (population > 1000) located in Shaanxi Province, which accounts for ~70% of musk deer breeding in China. A total of seven fecal samples were collected from musk deer that had recently received antibiotic treatments (antibiotic-exposed group, AE), and six samples were obtained from individuals that had not been exposed to antibiotics in the past six months (non-antibiotic-exposed group, non-AE). Musk deer were housed individually overnight and typically defecated around 6:00 a.m. Fecal samples were collected at 8:00 a.m. to ensure freshness and minimize environmental contamination. Musk deer do not exhibit coprophagic behavior. The primary antibiotics used in the management of musk deer included beta-lactams (e.g. cefalexin Tablets and cefixime tablets), sulfonamides (e.g. sulfaguanidine), aminoglycosides (e.g. amikacin sulfate), and macrolides (e.g. azithromycin). The non-antibiotic-exposed group had also undergone multiple antibiotic treatments more than six months prior to sample collection. In addition, six fecal samples were collected from wild musk deer inhabiting the Qinling Mountains, which constitute the largest natural habitat for this species. Fecal samples of wild musk deer were collected during field surveys guided by infrared camera monitoring and wildlife patrol records to ensure sampling within confirmed musk deer activity areas. Species identification was based on a combination of morphological, olfactory, and behavioral characteristics. Musk deer feces typically consist of small, granular pellets (0.3–0.4 cm in width, 0.5–0.6 cm in length), resembling peanut or wheat grains, with one end bluntly pointed and the other slightly indented or rounded. Fresh pellets are dark black with a mucous coating and dark green interior, becoming glossy upon drying. A distinctive musky odor, resulting from macrocyclic ketones in male secretions, further aided identification, whereas female pellets exhibited a typical herbivorous, grassy-fecal smell [18].

To assess potential transmission of ARGs from musk deer to humans, we collected seven fecal samples from farm workers who had been working on the farm for more than two years. For comparison, seven fecal samples were also collected from local villagers residing in the same community but with no history of exposure to the musk deer farm. All participants provided written informed consent prior to sample collection. Based on structured interviews, none reported antibiotic use within the six months preceding sampling. Fecal samples were collected on-site using sterile containers, immediately transported to the laboratory in a refrigerated container, and stored at −80°C until DNA extraction.

### DNA extraction and metagenomic sequencing

Fecal DNA was extracted using the Magnetic universal genomic DNA Extraction Kit (DP705) from TIANGEN Biotech (Beijing, China). Qualified DNA (concentration ≥ 10 ng/μL, A260/A280 ≥ 1.8 and A260/A230 ≥ 2.0) was sent to BMKGENE Co., Ltd. (Beijing, China) for metagenomic sequencing on the NovaSeq 6000 System (Illumina) with paired-end 150 bp reads.

### Collection of fecal metagenomes of wild and captive *Caprinae* species

To further evaluate the generality of captivity-associated shifts in the fecal resistome across animal species, we analyzed 69 fecal metagenomes from *Caprinae*, categorized into three groups: wild, zoo-captive, and farm-captive. Of these, 12 metagenomes from zoo-captive individuals were newly generated in this study, whereas the remaining 57 metagenomes were retrieved from publicly available databases. Specifically, the wild group consisted of 27 fecal metagenomes, comprising two impalas (*Aepyceros melampus*), three goats (*Capra hircus*), eight alpine ibexes (*Capra ibex*), two mouflons (*Ovis aries musimon*), one chinkara (*Gazella bennettii*), six goitered gazelles (*Gazella subgutturosa*), three sheep (*Ovis aries*), and two chamois (*Rupicapra rupicapra*). The zoo-captive group included a total of 14 samples, consisting of two publicly downloaded genomes (one goat and one sheep), along with 12 newly collected samples: three red lechwes (*Kobus leche*), two gorals (*Naemorhedus goral*), five argali (*Ovis ammon*), and two goats. The farm-captive group comprised 28 samples, including five goats and 23 sheep. Detailed information on all metagenomic datasets is provided in the Supplementary Table S1.

### Quality control, assembly, and binning of metagenomic data

Raw reads were processed using TRIMMOMATIC (v0.39) to remove adapters, primers, and low-quality reads (quality score < 15 or length < 36 bp) [19]. Host sequences were checked and removed using Kneaddata (v0.10.0) (http://huttenhower.Sph.Harvard.Edu/kneaddata). After quality control, non-host reads were randomly subsampled to a uniform sequencing depth of 10 Gb per sample using SeqKit (v2.10.1) [20].

Bacterial community structure was analyzed using PhyloFlash (v3.4) [21]. Assembly was performed using MEGAHIT (v1.2.9) with default parameters [22]. The MetaWRAP (v1.3.0) Binning module (-MetaBAT2, -MaxBIN2.0, and -CONCOCT) and Bin_refinement module were used to reconstruct the metagenomic-assembled genomes (MAGs) from all samples [23]. CoverM (v0.7.0) was used to calculate the proportion of metagenomic reads mapped to reconstructed MAGs in each sample. Mapping rates ranged from 31% to 50% across sample groups, with detailed results provided in Supplementary Table S2. CheckM (v1.2.2) was used to assess the quality of MAGs. Only MAGs with completeness ≥50% and contamination ≤10% were retained for further analyses [24]. Qualified MAGs were classified using the Genome Taxonomy Database Toolkit (GTDB-Tk, v2.4.0) [25].

### Identification of ARGs and mobile genetic elements

Clean reads were used to identify ARGs using ARGs-OAP (v3.2.2) [26], which classifies ARGs into types—corresponding to antibiotic classes (e.g. beta-lactam, tetracycline)—and subtypes, representing specific resistance genes (e.g. bla*NDM*, *tetM*). Subtypes within the same type often encode distinct enzymes that differ in their resistance mechanisms and substrate specificities. We considered an ARG present in a group only if it was detected in two or more samples from that group. Mobile genetic elements (MGEs) were identified using a self-constructed database [27]. The abundance of ARGs and MGEs was normalized to the copy number per cell [26]. DIAMOND (v2.0.6.144) was used to annotate the ARGs and MGEs located on contigs according to the criteria of identity ≥80% and gene coverage ≥80% [28].

### ARGs host, mobility analysis and health risk assessment

ARG-carrying contigs were used for classifying ARG hosts using CAT (v5.2.3) with default parameters [29]. The genetic location (chromosome, plasmid, and unclassified) of an ARG was predicted by PlasFlow (v3.5.4) [30]. Clinically important ARGs were identified according to Zhang *et al.* and Tarek *et al.* [31, 32], which considered the clinical availability of antibiotics, the mobility of ARGs, the pathogenicity of their hosts, and the potential for ARG transmission from environmental sources to humans. Potential human pathogenic bacteria were identified according to the pathogen list by the World Health Organization (WHO) and the Comprehensive Antibiotic Resistance Database (CARD) (https://card.mcmaster.ca/prevalence). The MetaCompare2.0 (v2.0) pipeline was applied to evaluate the human health resistome risk, referring to the potential of clinically relevant human pathogens to acquire ARGs, as well as the ecological resistome risk, which indicates the overall mobility and transferability of ARGs to pathogens [33].

### Prediction of horizontal gene transfer

MetaCHIP (v1.10.12) was used to predict HGT events and directions [34], by integrating sequence similarity alignment with taxonomic annotation of host bacteria. ARG-related HGT events were identified by annotating the predicted sequences using the SARG database (v3.2) through DIAMOND (v2.0.6.144). The identification of HGT events using MetaCHIP (v1.10.12) depended on reconstructing MAGs, a process that may result in the omission of HGT events that were not assigned to a MAG. To compensate for the issue, we also predicted HGT events on unbinned contigs. BEDTools (v2.31.1) was used to extract ARGs sequences with more than 80% completeness from contigs [35]. Multiple sequence alignment was performed using ClustalW, and the ARGs phylogenetic tree was constructed using MEGA11.0 by the maximum-likelihood (ML) method [36]. The presence of two identical ARGs on distinct bacterial hosts, at the species or higher rank levels, suggested potential HGT events between those bacterial hosts [37].

### Bacterial isolation and antibiotic susceptibility testing

Bacterial strains were isolated from the feces of antibiotic-exposed musk deer using MacConkey Agar, Brain Heart Infusion medium and Salmonella-Shigella agar. PCR was used to detect 15 clinically important ARGs and 15 virulence factors in the isolates, details regarding the primer sequences and annealing temperatures were provided in the Supplementary Materials and Supplementary Table S3.

Antibiotic susceptibility of *Escherichia coli* (*E. coli*) isolates was assessed using the Kirby-Bauer disk diffusion method. Details regarding the types and concentrations of antibiotics used are provided in the Supplementary Table S4. Briefly, bacterial suspensions were adjusted to a 0.5 McFarland standard and spread onto Mueller–Hinton agar plates. Antibiotic disks were placed on the agar surface, and plates were incubated at 37°C for 18 h. Inhibition zone diameters were measured and interpreted according to the Clinical and Laboratory Standards Institute (CLSI) M100 ED33 guidelines, with *E. coli* ATCC 43895 used as the quality control strain. Detailed protocols are available in the Supplementary Materials.

### Conjugation transfer assays

To assess the transfer potential of ARGs from the fecal microbiomes of antibiotic-exposed and non-exposed musk deer, filter mating assays were performed. Fecal bacterial suspensions or isolated *E. coli* strains served as donors, and a sodium azide– and kanamycin-resistant *E. coli* G53 strain as the recipient. Donor and recipient cells were mixed at a 1:1 ratio, spotted onto 0.22-μm nitrocellulose filters placed on LB agar, and co-cultured at 37°C for 48 h. Transconjugants were selected on LB agar containing sodium azide, kanamycin, and one of four antibiotics: chlortetracycline, ceftiofur, gentamicin, or ciprofloxacin. Conjugation frequency was calculated as the number of transconjugants per recipient cell. All assays were performed in triplicate; detailed protocols are provided in the Supplementary Materials.

### Statistical analysis

The phylogenetic tree of MAGs was constructed using GTDB-Tk and visualized using iTOL (https://itol.embl.de). Alpha diversity index, distance matrix, constrained principal coordinates analysis (CPCoA), Venn diagrams, and stacked bar charts were generated using the ImageGP (https://www.bic.ac.cn). Co-occurrence networks of ARGs and MGEs were constructed using Cytoscape software [38]. Data were analyzed using GraphPad Prism 9 software [39]. For statistical analysis, differences in ARG abundance, MGE abundance, taxonomic composition, resistome risk scores, and conjugation frequency were evaluated using the Kruskal-Wallis test, followed by Mann–Whitney U tests for pairwise comparisons. *P-*values were adjusted using the Benjamini-Hochberg procedure to control the false discovery rate. Differences in resistome composition were evaluated using CPCoA based on Bayesian posterior-derived dissimilarity matrices, with significance tested using 999 permutations.

## Results

### Captive breeding greatly expands the fecal resistome of musk deer and changes microbiome compared to wild individuals

**Figure 1:**
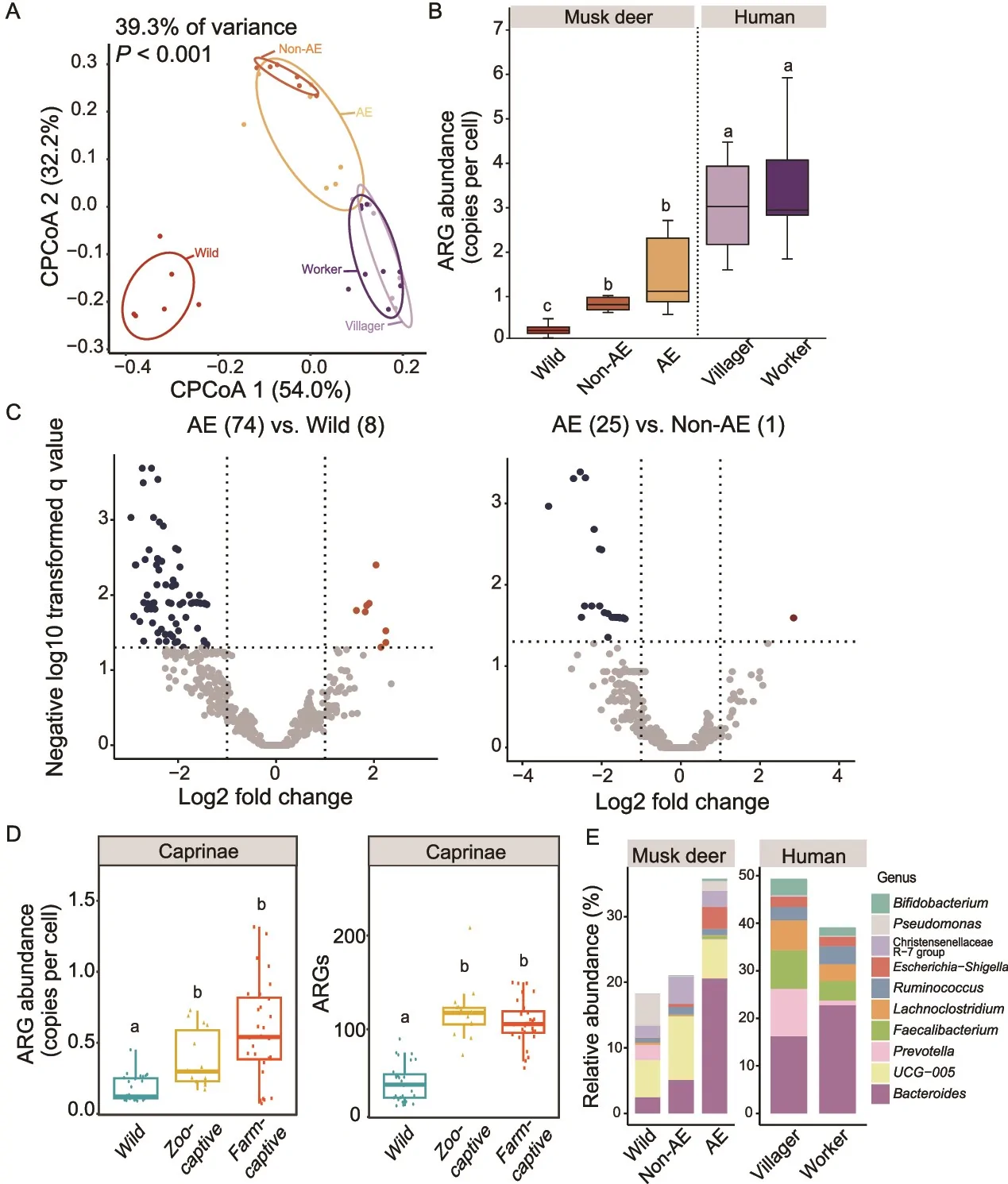
Fecal resistome and microbiome in musk deer, *Caprinae* species and farm workers. (A) Constrained principal coordinates analysis (CPCoA) plot showing the separation of resistome profiles across groups. (B) Total abundance of ARGs. (C) Volcano plots showing ARGs with higher abundance in captive musk deer compared to the wild group; numbers in paratheses indicate the number of significantly higher-abundance ARGs in each group. (D) Total ARG abundance (left) and the number of detected ARGs (right) in *Caprinae* feces under wild, zoo-captive and farm-captive conditions. (E) Relative abundance of the top 10 bacterial genera across groups. AE: Musk deer with recent antibiotic exposure; non-AE: musk deer without antibiotic exposure in the past six months. Different lowercase letters indicate significant differences between groups (*P <* .05, Mann–Whitney U test). Groups sharing the same letter are not significantly different. Resistome composition differences were assessed using CPCoA based on Bayesian posterior-derived dissimilarity matrices, with significance tested by permutation (999 permutations).

Significant differences were observed in the resistome profiles between wild and captive musk deer, with notable similarities between antibiotic-exposed and non-antibiotic-exposed captive musk deer (*P <* .05, Fig. 1A). A total of 25 types encompassing 672 subtypes of ARGs were detected in musk deer feces. More ARG subtypes were identified in the feces of antibiotic-exposed musk deer (504 subtypes) compared to those without antibiotic exposure (321 subtypes) and wild musk deer (322 subtypes). The total abundance of ARGs in antibiotic-exposed musk deer (1.55 copies per cell) was 5.03-fold higher than in wild musk deer and 1.86-fold higher than in non-antibiotic-exposed musk deer (Fig. 1B and Supplementary Fig. S1). Antibiotic-exposed musk deer exhibited higher abundances of 74 ARG subtypes in their fecal microbiomes compared to wild musk deer, and 25 subtypes compared to non-antibiotic-exposed musk deer (Fig. 1C). These ARGs with elevated abundance were consistent with the antibiotics used in captive breeding practices. 147 unique ARGs were found only in antibiotic-exposed musk deer, far exceeding the 7 in non-antibiotic-exposed groups (Supplementary Fig. S2A). Similarly, both captive *Caprinae* species groups exhibited a significantly higher resistome abundance compared to the wild group (0.17 copies per cell), with the farm-captive group (0.58 copies per cell) showing a higher abundance than the zoo-captive group (0.38 copies per cell) (Fig. 1D). No statistically significant difference was observed between the farm-captive and zoo-captive groups (Fig. 1D), but both captive groups carried a significantly greater ARG diversity than the wild group (*P <* .05).

Significant differences were observed in the fecal microbiome among wild musk deer, antibiotic-exposed musk deer, and non-antibiotic-exposed musk deer (*P <* .05, Supplementary Fig. S3A). The abundance of *Bacteroidaceae* in antibiotic-exposed musk deer was 20.6%, significantly higher than the 5.1% observed in non-antibiotic-exposed musk deer (*P <* .05, Fig. 1F). The abundance of potential pathogens such as *Bacteroides* and *Escherichia-Shigella* also increased significantly in antibiotic-exposed musk deer (*P <* .05). Procrustes analysis confirmed that the resistome profile was significantly associated with microbiome composition (*M^2^* = 3.36, *P <* .001, Supplementary Fig. S3B). These findings collectively indicate that captivity and antibiotic exposure are associated with distinct shifts in the fecal microbiome and resistome of musk deer.

### Exposure to clinically important antibiotics increased fecal resistome risks in musk deer

**Figure 2:**
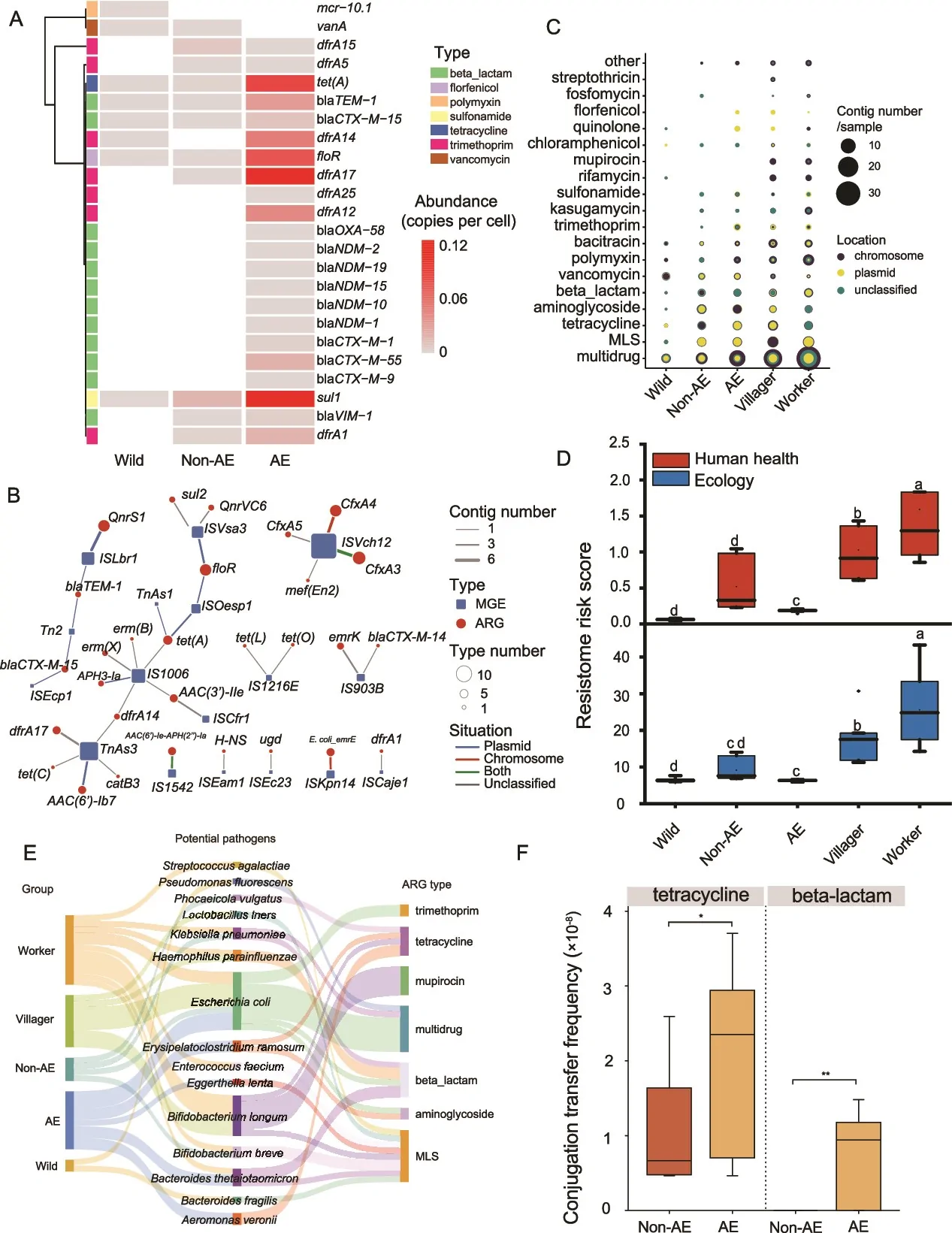
Impact of human antibiotic use on the health risks of antibiotic resistome in musk deer and farm workers. (A) Abundance of clinically important ARGs across groups. (B) Co-occurrence network of ARGs and MGEs. (C) Genetic locations of different types of ARGs. (D) Resistome risk score assessed using MetaCompare2. Human health risk reflects the potential for ARGs to be acquired by clinically relevant pathogens; and ecological risk indicates the overall mobility and transferability of ARGs based on their association with MGEs. (E) Detection of potential pathogens carrying ARGs across groups. (F) Conjugation transfer frequencies in fecal samples from captive musk deer. Different lowercase letters in the same color indicate significant difference (*P <* .05, Kruskal-Wallis test). Asterisks indicate statistically significant differences between pairs of groups (*P <* .05, Mann–Whitney U test). (^*^  *P <* .05, ^**^  *P <* .01) AE: musk deer with recent antibiotic exposure; non-AE: musk deer without antibiotic exposure in the past six months.

Clinically important ARGs were widely detected in the fecal microbiomes of captive musk deer. Twenty-eight clinically important ARGs, including *blaNDM-1*, *blamcr-10.1*, *blaTEM-1*, *dfrA17*, *floR*, and *tetA* were detected in feces from captive musk deer. The abundance of clinically important ARGs showed a clear gradient across groups (Fig. 2A), with the highest level in the antibiotic-exposed group (0.087 copies per cell), followed by the non-exposed group (0.0055 copies per cell), and the lowest in the wild group (0.00042 copies per cell).

The total MGE abundance in antibiotic-exposed musk deer group was also the highest, reaching 0.73 copies per cell, which was 16.3-fold higher than that in non-antibiotic-exposed musk deer (0.042 copies per cell) and 44.9-fold higher than in wild musk deer (0.015 copies per cell) (Supplementary Fig. S4). The abundance of integrons in antibiotic-exposed group was also significantly higher compared to both non-antibiotic-exposed and wild musk deer groups (*P <* .05, Supplementary Fig. S4). Certain associations between MGEs and ARGs were observed, e.g. *aac(6′)-Ie-aph(2″)-Ia* was associated with *IS1542*, *E. coli emrE* with *ISKpn14* (Fig. 2B). ARGs associated with the banned veterinary antibiotic florfenicol (*floR* and *pp-flo*) were detected exclusively on plasmids from samples of antibiotic-exposed musk deer, farm workers, and villagers (Fig. 2C and Supplementary Fig. S5).

We detected 10 ARG-carrying pathogens in musk deer fecal samples, with the highest detection rate observed in the antibiotic-exposed musk deer group, followed by the non-antibiotic-exposed group, and the wild group had the least detection rate. The ARGs carried by pathogens in musk deer predominantly confer multidrug resistance and tetracycline resistance (Fig. 2E). The antibiotic-exposed group showed significantly higher human health- and ecological-resistome risk scores than the non-antibiotic-exposed and wild groups (*P <* .05, Fig. 2D).

To evaluate the horizontal transfer potential of ARGs in the fecal microbiomes of captive musk deer, conjugation assays were conducted using fecal suspensions from antibiotic-exposed (AE) and non-antibiotic-exposed (non-AE) individuals. Antibiotic exposure significantly enhanced conjugative transfer of ARGs in the fecal microbiomes: the transfer frequencies of tetracycline resistance genes (2.1 × 10^−8^) and beta-lactam resistance genes (7.6 × 10^−9^) in the AE group were significantly higher than those in the Non-AE group (1.0 × 10^−8^ and 0, respectively; *P <* .05; Fig. 2F).

### Clinically important ARGs are primarily associated with *Enterobacteriaceae* in the fecal microbiome of captive musk deer

**Figure 3:**
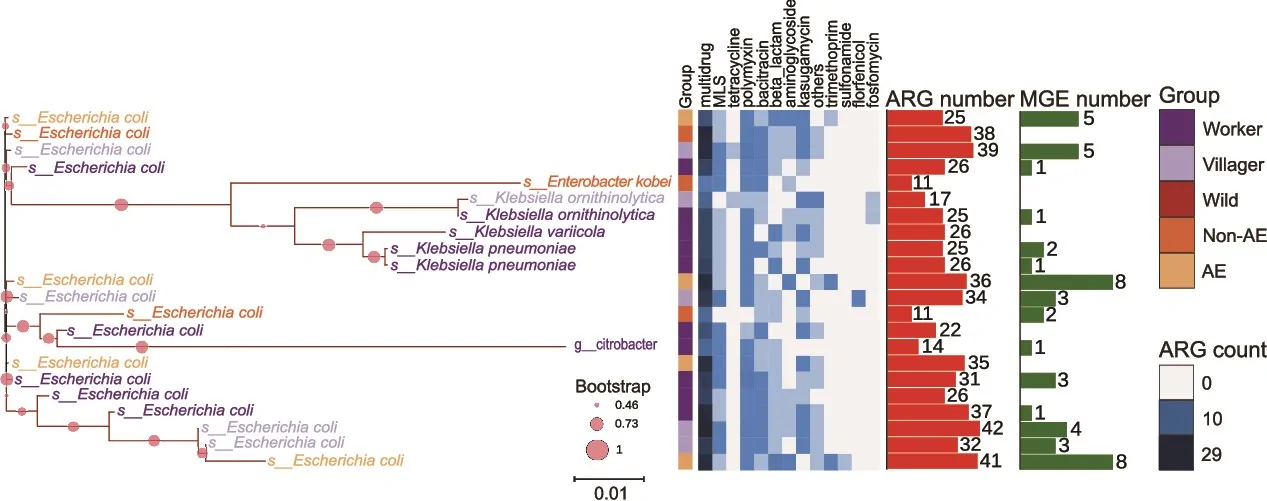
Phylogenetic tree of *Enterobacteriaceae* metagenome-assembled genomes (MAGs) reconstructed from musk deer, villager, and farm worker fecal metagenomes, showing their associated ARGs and MGEs. The blue heatmap indicates the number of various types of ARGs detected in each MAG. Red and green bars represent the total number of ARGs and MGEs detected per MAG, respectively. The scale bar represents 0.01 substitutions per site. AE: musk deer with recent antibiotic exposure; non-AE: musk deer without antibiotic exposure in the past six months.

To identify the bacterial hosts of ARGs and characterize their genetic context, we combined MAGs with culture-based isolation to analyze ARG carriage in the fecal microbiomes of musk deer and farm workers. We reconstructed 1528 MAGs from the fecal metagenomes of wild musk deer, captive musk deer, farm workers, and villagers not exposed to musk deer farm. Among them, 194 MAGs carried 135 distinct ARG subtypes, potentially conferring resistance to 18 classes of antibiotics. The predominant resistance types included multidrug, MLS, tetracycline, and polymyxin resistance genes (Supplementary Fig. S6). Among them, 60 MAGs harbored 2–10 ARG subtypes, and 22 MAGs carried more than 10 ARG subtypes. These 22 high-ARG-load MAGs all belonged to the family *Enterobacteriaceae* and collectively carried 74 ARG subtypes, most of which were multidrug resistant genes. These ARGs were frequently flanked by MGEs (Fig. 3). One *Enterobacteriaceae* MAG reconstructed from antibiotic-exposed musk deer group carried 41 ARGs, including three clinically important ARGs: *blaCTX-M-15*, *dfrA17*, and *blaTEM-1*. *E. coli* MAGs assembled from captive musk deer and human feces (farm workers and villagers) also harbored various ARGs, whereas no ARGs were detected in *E. coli* MAGs derived from wild musk deer.

**Figure 4:**
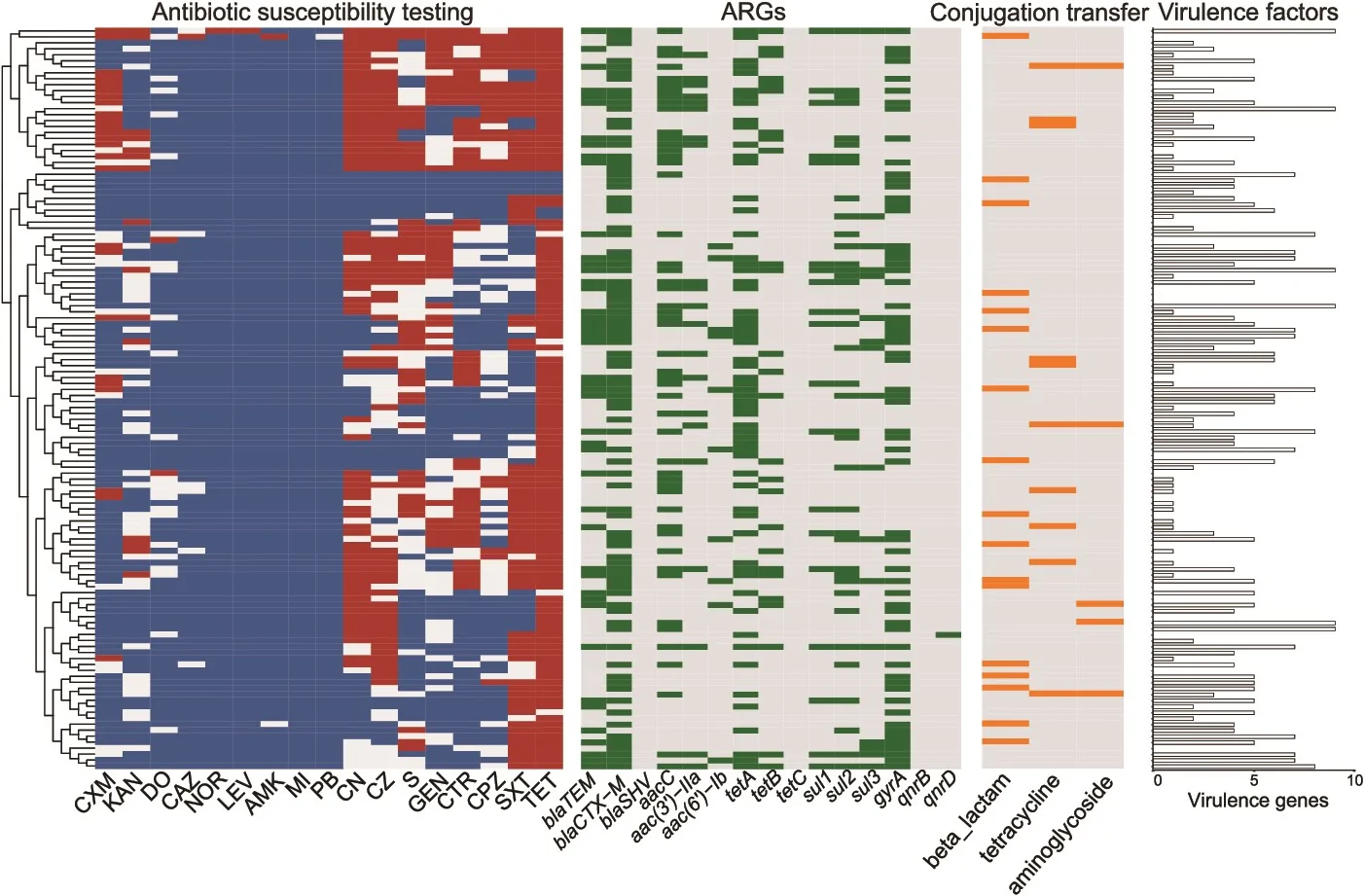
Antibiotic susceptibility, ARG, conjugation transfer, and virulence factor detection in *Escherichia coli*. Blue boxes indicate susceptible, grey box indicates intermediate, and red box indicates resistant. Green boxes indicate the presence of corresponding ARGs. Orange boxes indicate the occurrence of conjugation transfer. CN: Cefalexin; CZ: Cefazolin; CXM: Cefuroxime sodium; CAZ: Ceftazidime; CTR: Ceftriaxone sodium; CPZ: Cefoperazone; AMK: Amikacin; GEN: Gentamicin; KAN: Kanamycin; S: Streptomycin; TET: Tetracycline; MI: Minocycline; DO: Doxycycline; NOR: Norfloxacin; LEV: Levofloxacin; SXT: Cotrimoxazole; PB: Polymyxin B.

A total of 265 bacterial strains were isolated from fecal samples of antibiotic-exposed musk deer, including 124 *E. coli* strains, 29 *Shigella flexneri* strains, 21 *Klebsiella pneumoniae* strains, and other species (Supplementary Fig. S7). All *E. coli* isolates were analyzed for the presence of ARGs, virulence factors, and antibiotic susceptibility profiles. 111 *E. coli* strains carried ARGs, with 70% harboring beta-lactam resistance genes *blaCTX-M* and 50% carrying tetracycline resistance gene *tetA*. Three *E. coli* isolates carry 10 ARG subtypes, conferring resistance to multiple antibiotic classes, including beta-lactams, aminoglycosides, tetracyclines, quinolones, and sulfonamides (Fig. 4). Ninety-five of the ARG-carrying strains also possessed virulence factors, with ~70% containing more than three virulence factors. The predominant virulence factors identified were *PhoA* (65 strains), *OmpA* (58 strains), *Afa* (56 strains), *ECS3703* (47 strains), and *FimA* (41 strains) (Fig. 4 and Supplementary Fig. S8). Phenotypic antimicrobial susceptibility testing revealed that 33 *E. coli* isolates exhibited resistance to more than five out of 17 tested antibiotics. Strain ZD4–8 was resistant to as many as 12 antibiotics. More than 90% of *E. coli* isolates were resistant to tetracycline (Fig. 4), whereas all isolates remained susceptible to minocycline and the polypeptide antibiotic polymyxin B. We then experimentally validated the transferability of ARGs in *E. coli* isolates. Of the tested strains, 34 exhibited the capacity to transfer plasmids to recipient cells, thereby mediating the horizontal dissemination of the ARGs they carry (Fig. 4).

### ARG transmission between the fecal microbiomes of captive musk deer and farm workers

To evaluate the potential spread of ARGs from captive musk deer to farm workers, we compared fecal ARG profiles between farm workers and local villagers without farm exposure. The feces of farm workers exhibited a great diversity and abundance of ARGs, with 217 subtypes detected at an average of 3.63 copies per cell. In contrast, villagers not exposed to musk deer farm had 195 subtypes with an abundance of 3.05 copies per cell (Fig. 1B and Supplementary Fig. S2B). Total of 52 unique ARGs were detected in farm workers, higher than the 30 in villagers (Supplementary Fig. S2A). A total of 170 ARG subtypes were shared between captive musk deer and farm workers. Among the 52 ARGs unique in workers relative to villagers, 44 were also identified in musk deer samples, with an average relative abundance of 3.5%. Moreover, the fecal resistome of farm workers showed significantly higher risk scores compared to that of local villagers who had never been exposed to the musk deer farm (*P <* .05, Fig. 2D).

**Figure 5:**
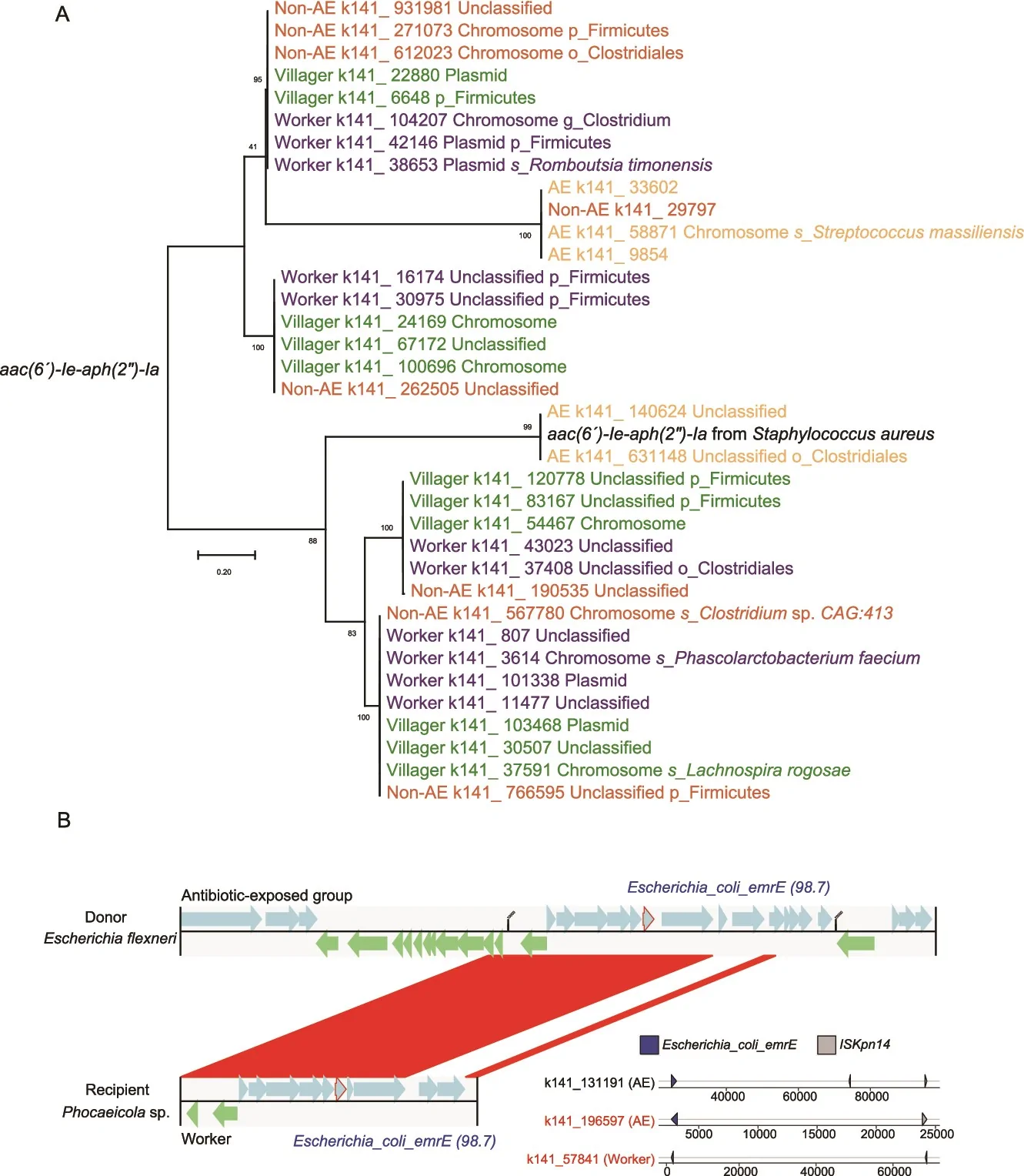
Transmission of ARGs between captive musk deer and farm workers. (A) Maximum likelihood phylogenetic tree of the *aac(6′)-Ie-aph(2″)-Ia* gene. The italicized black sequence was retrieved from the SARG database. (B) Evidence of horizontal gene transfer of the *Escherichia_coli_emrE* gene. Left: HGT event predicted by MetaCHIP; right: co-occurrence of MGEs flanking the gene. AE: musk deer with recent antibiotic exposure; non-AE: musk deer without antibiotic exposure in the past six months.

We identified 522 HGT events between bacterial populations associated with humans and captive musk deer, involving 4981 genes. Among these cross-species HGT events, the number of transfer events between antibiotic-exposed and farm workers was much higher (163 events, 2093 genes) compared to those between non-antibiotic-exposed and farm workers (74 events, 576 genes). The same *aac(6′)-Ie-aph(2″)-Ia* gene sequence was identified across multiple bacterial species (*Romboutsia timonensis*, *Streptococcus massiliensis*, *Lachnospira rogosae* and *Phascolarctobacterium faecium*) in both captive musk deer and farm worker fecal samples (Fig. 5A and Supplementary Fig. S9). Additionally, HGT prediction analysis revealed that *S. flexneri* served as a potential donor of the resistance gene *Escherichia_coli_emrE*, suggesting its capacity to transfer this gene from antibiotic-exposed musk deer to the recipient bacterium *Phocaeicola* sp. in farm workers (Fig. 5B and Supplementary Fig. S9).

## Discussion

Our study reveals a previously overlooked pathway for the enrichment and dissemination of ARGs: the captive breeding of specialty animals. We found that captivity significantly reshapes and expands the fecal resistome of musk deer (*M. berezovskii*). Compared to their wild counterparts, fecal bacteria of captive musk deer exhibited higher diversity and abundance of ARGs. The shift and expansion in the fecal resistome may be attributed to various factors associated with captivity, including high population density, artificial diets, antibiotic use, and increased human contact [9, 40]. Similarly, previous studies have shown that domestication profoundly alters the gut microbiome of yak (*Bos grunniens*), chimpanzee (*Pan troglodytes*), gorilla (*Gorilla gorilla*), and cynomolgus macaque (*Macaca fascicularis*) [9, 40, 41], where bacterial composition plays a key role in shaping the resistome. Concerningly, when these captive animals re-enter the wild (e.g. through escape or release), they may spread their acquired antibiotic-resistant bacteria (ARB) and ARGs to wild populations and predators, accelerating the spread of antibiotic resistance in natural environments [42]. Moreover, captivity is widely used for wildlife breeding, conservation, and tourism, all of which may lead to the expansion of animal associated resistomes. Through a comprehensive analysis of 69 fecal metagenomes of wild and captive *Caprinae* species, we demonstrate that captivity (both zoo-captive and farm-captive conditions) consistently boost both the diversity and abundance of the fecal resistome across animal species.

We observed elevated potential health risks associated with the fecal resistome of musk deer following the use of clinically important antibiotics. Compared to musk deer without antibiotic exposure in the past six months, those recently treated exhibited higher abundances of 25 clinically important ARGs in their fecal microbiomes, including *blaNDM-1*, *blaCTX-M-30*, and *tetA*, which closely correspond to the antibiotics commonly used in musk deer therapy. For example, trimethoprim is commonly co-administered with sulfamethoxazole to treat gastrointestinal infections in musk deer [43]. This practice likely contributes directly to the accumulation of sulfonamide-related resistance genes in the fecal microbiome [44]. In specialty animal farming, particularly for species with short domestication history and undeveloped breeding techniques, animals are more susceptible to bacterial infections, which has led to widespread overuse and misuse of clinically important antibiotics [13, 14]. MetaCompare2 analysis further indicates that musk deer recently exposed to antibiotics pose significantly higher risks for humans (potential for human pathogens of acute resistance concern to acquire ARGs) and for ecological dissemination (overall mobility of ARGs and potential for pathogen acquisition), compared to those not recently exposed and wild counterparts [33]. Therefore, given the rapid global growth of specialty animal farming, there is an urgent need to develop tailored breeding approaches and strengthen regulation of associated practices.

We observed that ARGs present in the fecal microbiome of captive musk deer were largely associated with members of *Enterobacteriaceae*, particularly *E. coli*. As a leading cause of diarrheal disease in musk deer, *E. coli* also represents a common commensal and opportunistic pathogen in both humans and other animal species [45]. Moreover, its wide ecological versatility enables it to act as a critical vector in the interspecies transmission of ARGs among animals, humans, and environmental reservoirs [46, 47]. We identified several MGE-associated ARGs in *E. coli* MAGs from musk deer with antibiotic exposure, in contrast, no ARGs were detected in the *E. coli* MAGs derived from wild musk deer. To complement our bioinformatic findings, we isolated 124 *E. coli* strains from the feces of musk deer recently treated with antibiotics and characterized their ARGs, virulence genes, antibiotic susceptibility profiles, and conjugative ARG transfer capabilities. The results revealed that these isolates carry multiple clinically important ARGs (e.g. *blaTEM* and *blaCTX-M*) and virulence factors, and exhibit resistance to up to 12 different antibiotics. These findings provide strong experimental validation for the conclusions derived from our bioinformatic analyses, reinforcing the critical role of captive breeding systems in the dissemination of clinically relevant ARGs.

Our study provides evidence suggestive of ARG exchange between musk deer and farm workers through bacterial colonization and HGT within their associated microbiomes. Farm workers harbored significantly higher abundance and diversity of ARGs compared to villagers with similar lifestyles but no exposure to musk deer farms. Moreover, workers shared more ARGs with musk deer than did villagers. Among the 52 ARGs unique in workers relative to villagers, 44 were also detected in musk deer samples with an average relative abundance of 3.5%, supporting the potential for cross-host ARG spillover between musk deer and workers. In addition, 26 ARGs with significantly higher abundance in workers relative to villagers were not detected in musk deer samples, suggesting that occupational exposures such as antibiotics and disinfectants may also shape the workers’ resistomes.

We inferred over 500 putative HGT events between bacterial populations of captive musk deer and farm workers, with significantly more transfers involving antibiotic-exposed deer than non-exposed individuals. Additionally, identical ARB were detected in both farm workers and captive musk deer but not in villagers, implying possible colonization of workers’ gut microbiota by musk deer derived ARB. We identified identical ARG sequences carried by distinct bacterial taxa present in fecal microbiomes of both musk deer and farm workers, indicating potential HGT of ARGs across the animal–human interface. These findings align with previous studies showing that exposure to livestock farms significantly increases the resistome abundance in farm workers and veterinary students [48–50]. Collectively, our results highlight occupational contact as a critical route for ARG dissemination between farm- and human-associated microbiomes, underscoring the urgent need for improved biosecurity practices and antibiotic stewardship in farming systems. Nevertheless, current evidence for ARG transmission at the animal–human interface remains largely bioinformatic and requires further experimental validation. Moreover, the resistome risks estimated by computational method represent theoretical potentials based on genomic co-occurrence; they do not account for actual exposure levels, functional gene expression, or ecological compatibility with the human microbiome—factors essential for realistic risk quantification. Although such predictions may inform preliminary hazard identification, their integration into quantitative microbial risk assessment (QMRA) frameworks remains limited without empirical exposure data and validation of transfer functionality.

In conclusion, this study suggests that captivity may play a previously underappreciated role in shaping and facilitating the spread of ARGs in wildlife, and points to potential dissemination of ARG-carrying bacteria and HGT events at the animal–human interface. Given the rapid global expansion of specialty animal farming, we strongly advocate for increased attention and regulation of captive breeding practices, which is crucial for both wildlife conservation and global public health.

## Supplementary Material

Supplementary_materials_wrag009

## Data Availability

The raw metagenomic sequencing data were deposited in the NCBI database under accession number PRJNA1135790 and in the CNCB database under accession number PRJCA041365.
