# Supplementary material for: Captive breeding of specialty animals represents an overlooked yet critical reservoir for spreading antibiotic resistance genes
Source: ISME J. 2026 Jan 28;20(1):wrag009. doi: 10.1093/ismejo/wrag009 (PMC12904276; doi:10.1093/ismejo/wrag009)
Supplement: Supplementary_materials_wrag009 [file supplementary_materials_wrag009.docx]

Captive breeding of specialty animals represents an overlooked yet critical reservoir for spreading antibiotic resistance genes

Jiao Xi^1,2#^, Hanxiang Tao^1,2#^, Ziyi Zhang^1,2^, Biyao Lian^1,2^, Wei Sun^1,2^, Yang Zhang^1,2^, Shuhai Bu^3^*, Xiaojun Yang^4^*, Xun Qian^1,2^*

1 College of Natural Resources and Environment, Northwest A&F University, Yangling, Shaanxi 712100, China

2 Interdisciplinary Research Center for Soil Microbial Ecology and Land Sustainable Productivity in Dry Areas, Northwest A&F University, Yangling, Shaanxi 712100, China

3 College of Life Sciences, Northwest A&F University, Yangling, Shaanxi, 712100, China

4 College of Animal Science and Technology, Northwest A&F University, Yangling, Shaanxi, 712100, China.

#These authors contributed equally: Jiao Xi and Hanxiang Tao.

*Corresponding author: Xun Qian (qianxun@nwafu.edu.cn)

**Supplementary Materials**

**Bacterial isolation and antibiotic susceptibility testing**

Bacterial strains were isolated from the feces of antibiotic-exposed musk deer using MacConkey Agar, Brain Heart Infusion medium and Salmonella-Shigella agar. PCR was used to detect 15 clinically important ARGs and 15 virulence factors in the isolates, details regarding the primer sequences and annealing temperatures were provided in the Supplementary Table S3. Each PCR amplification was prepared with a mixture containing 10 µL of 2× Taq PCR Master Mix (Qiagen, Hilden, Germany), 1 µL each of 10 µM forward and reverse primers, 6 µL ddH₂O, and 2 µL of DNA template. Subsequently, the PCR amplification products were analyzed using electrophoresis on 1% agarose gels.

Antibiotic susceptibility of *Escherichia coli* (*E. coli*) isolates was assessed using the Kirby-Bauer disk diffusion method. Details regarding the types and concentrations of antibiotics used are provided in the Supplementary Table S4. Briefly, bacterial strains were first cultured in Mueller-Hinton broth at 37°C with shaking at 180 rpm for 20 hours. Following incubation, cultures were adjusted to a 0.5 McFarland standard (approximately 1.5 × 10⁸ CFU/mL) by dilution in sterile 0.9% NaCl solution. A 100 µL aliquot of each suspension was evenly spread onto Mueller-Hinton agar plates. Antibiotic discs (BioDee Biotechnology Co., Ltd., Beijing, China) were placed on the agar surface with a minimum distance of 15 mm from the plate edge and 24 mm between discs. A total of 17 antibiotics representing six major classes were used for the testing: beta-lactams, aminoglycosides, tetracyclines, quinolones, sulfonamides, and polypeptides. After incubation at 37°C for 18 hours, inhibition zone diameters were measured using a vernier caliper. Susceptibility profiles were classified as susceptible (S), intermediate (I), or resistant (R) according to the M100 ED33 guidelines of the Clinical and Laboratory Standards Institute (CLSI). *E. coli* ATCC 43895 was used as the reference strain throughout the testing process.

**Community-wide level conjugation transfer assays**

We conducted the filter mating assays to assess the actual transfer potential of ARGs from the native microbial communities in antibiotic-exposed and non-antibiotic-exposed groups. The bacterial suspensions from these fecal samples as the donors, whereas sodium azide- and kanamycin-resistant E. coli G53 was used as the recipient. Donor and recipient suspensions were mixed at a 1:1 ratio in Phosphate Buffered Saline (PBS). The mixture was transferred onto sterile nitrocellulose filters (0.22 μm pore size) placed on LB agar plates and co-cultured at 37°C for 48 h. Control plates contained donor or recipient cells alone. After incubation, transconjugants were recovered by washing with PBS, serially diluted, and plated on LB agar supplemented with sodium azide, kanamycin, and one of the following selective antibiotics to select for transferred ARGs: chlortetracycline (a tetracycline-class antibiotic, 16 µg/mL), ceftiofur (a beta-lactam antibiotic, 20 µg/mL), gentamicin (an aminoglycoside, 20 µg/mL), or ciprofloxacin (a fluoroquinolone, 20 µg/mL). In parallel, conjugation assays were also performed using individual *E. coli* isolates recovered from fecal samples as donors. These isolates were cultured in LB broth overnight and mixed with the same recipient strain (*E. coli* G53) at a 1:1 ratio in PBS. The subsequent steps were identical to those described above for community-level mating assays. All experiments were performed in triplicate. Conjugation transfer frequency was calculated as the number of transconjugants per recipient cell. Transconjugants were confirmed by fluorescence and resistance profiling.

**Supplementary Figures**


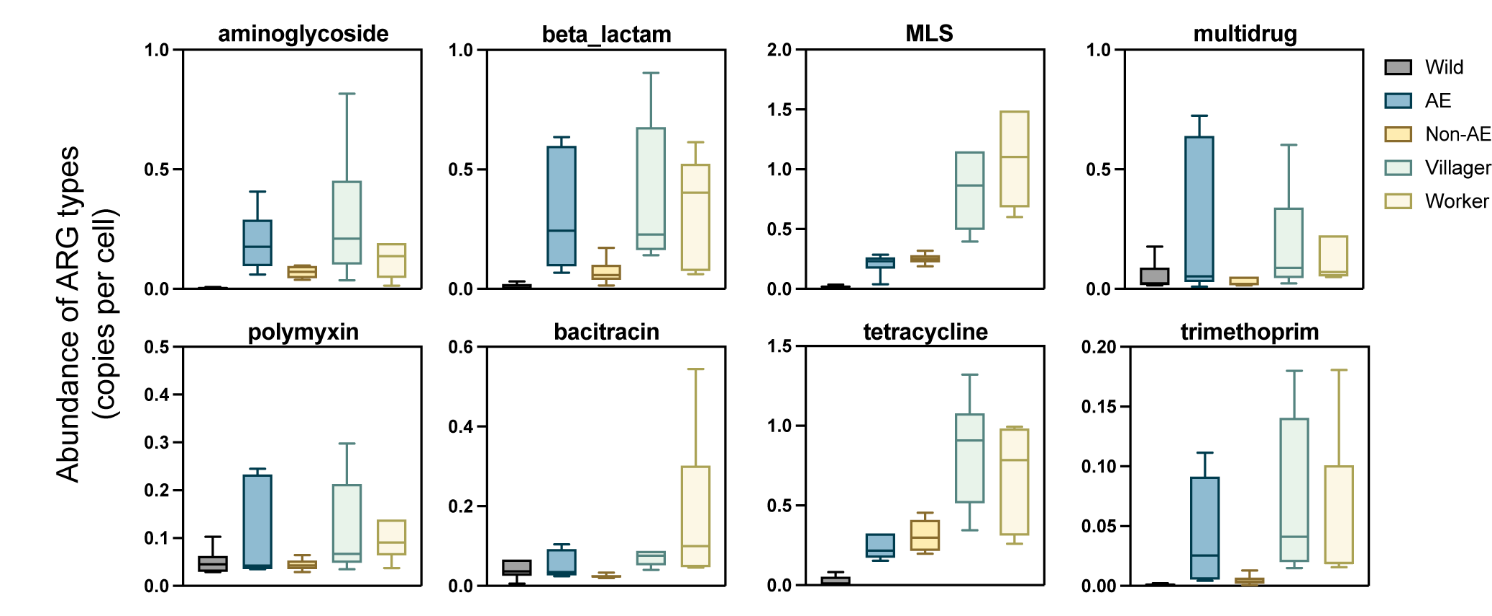


**Fig.S1** Abundance of the top eight ARG types. **AE**: musk deer with recent antibiotic exposure; **Non-AE**: musk deer without antibiotic exposure in the past six months.


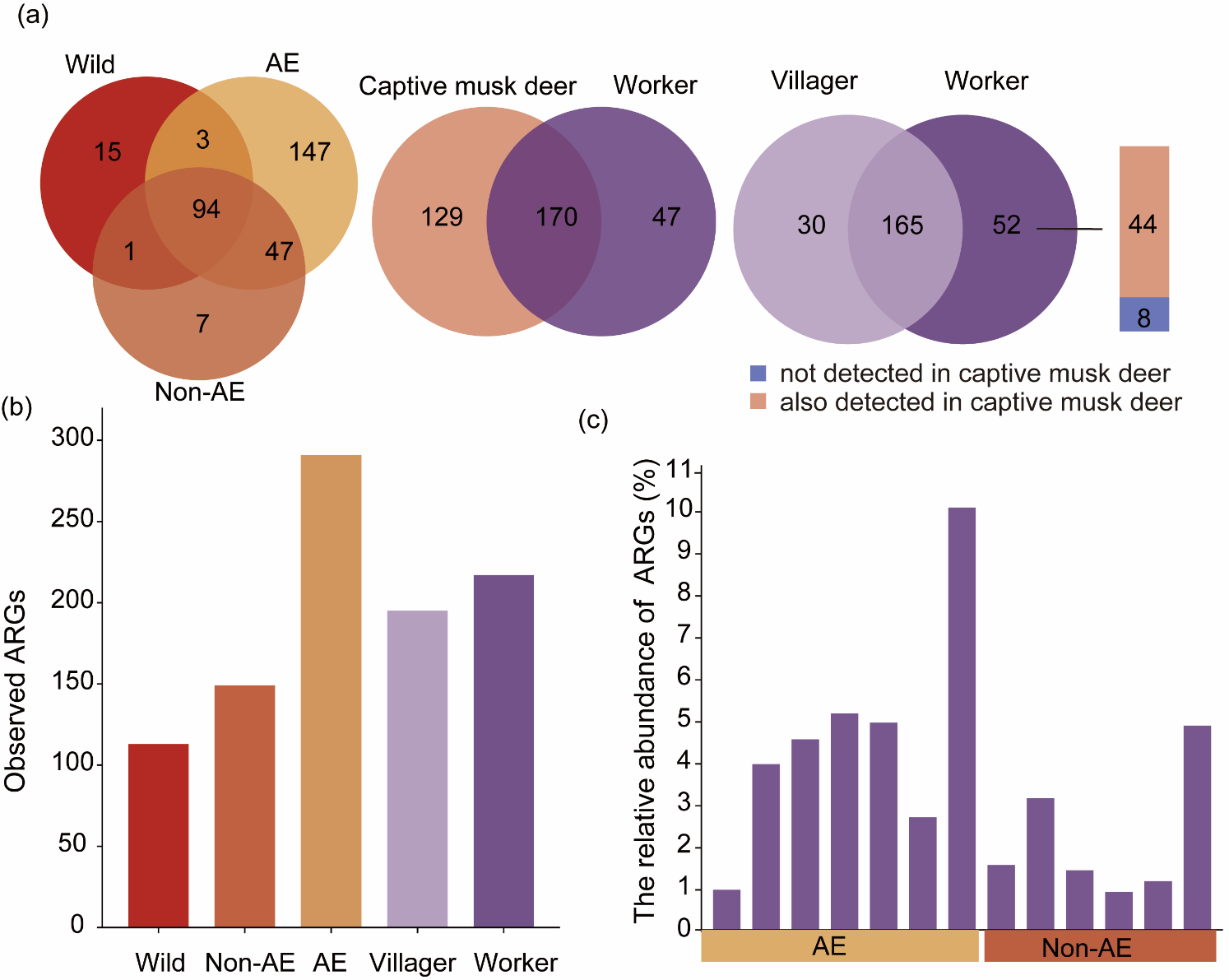


**Fig.S2** Shared and unique ARGs among musk deer and human fecal samples. (a) Venn diagrams illustrate pairwise ARG sharing among wild, AE, and Non-AE, between workers and captive musk deer (AE + Non-AE), and between villagers and workers. (b) The number of ARGs observed in each group. (c) Relative abundance of ARGs that only detected in workers within the fecal resistomes of captive musk deer. **AE**: musk deer with recent antibiotic exposure; **Non-AE**: musk deer without antibiotic exposure in the past six months.


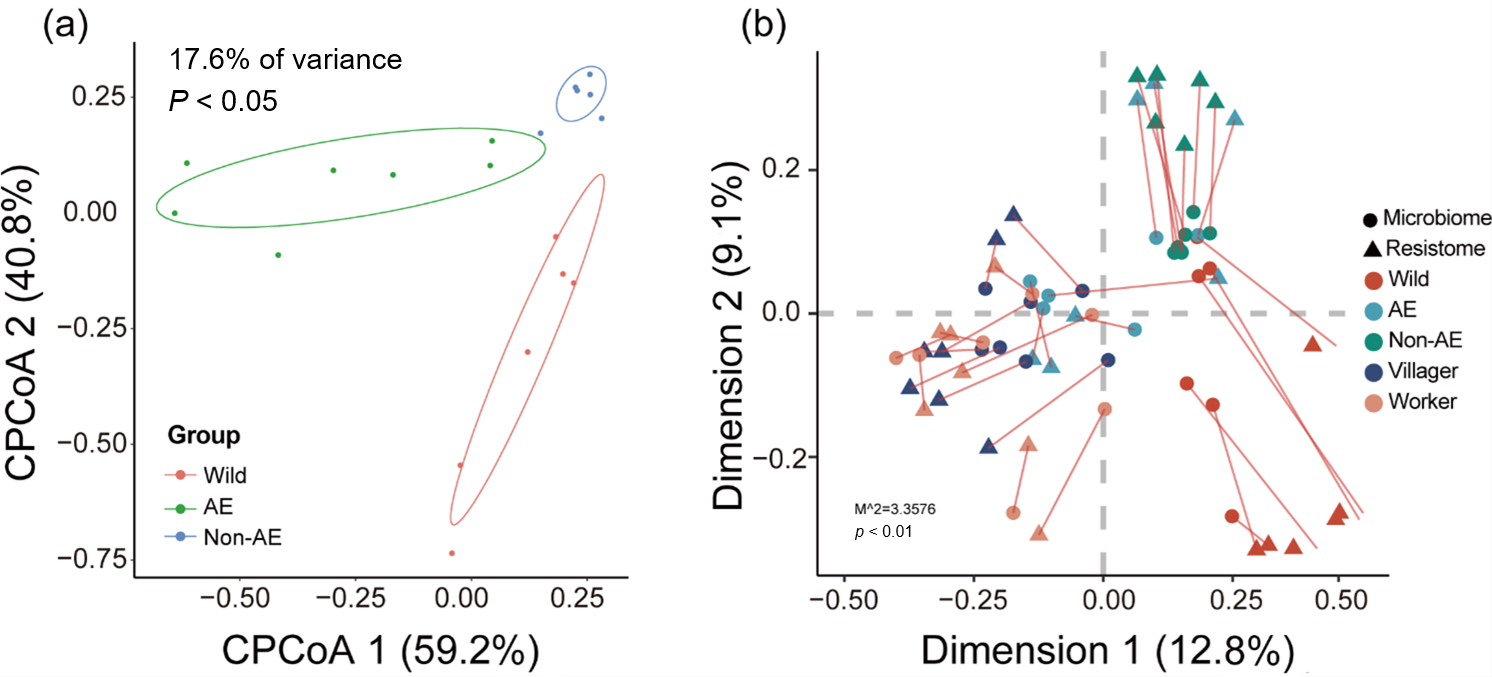


**Fig.****S3** Bacterial community composition in musk deer and human feces. (a) Constrained principal coordinates analysis (CPCoA) plot showing the segregation of fecal microbiomes among the antibiotic-exposed (**AE**), non-antibiotic-exposed (**Non-AE**), and wild musk deer groups. (b) Procrustes analysis showing the relationship between ARG profile and microbial composition.


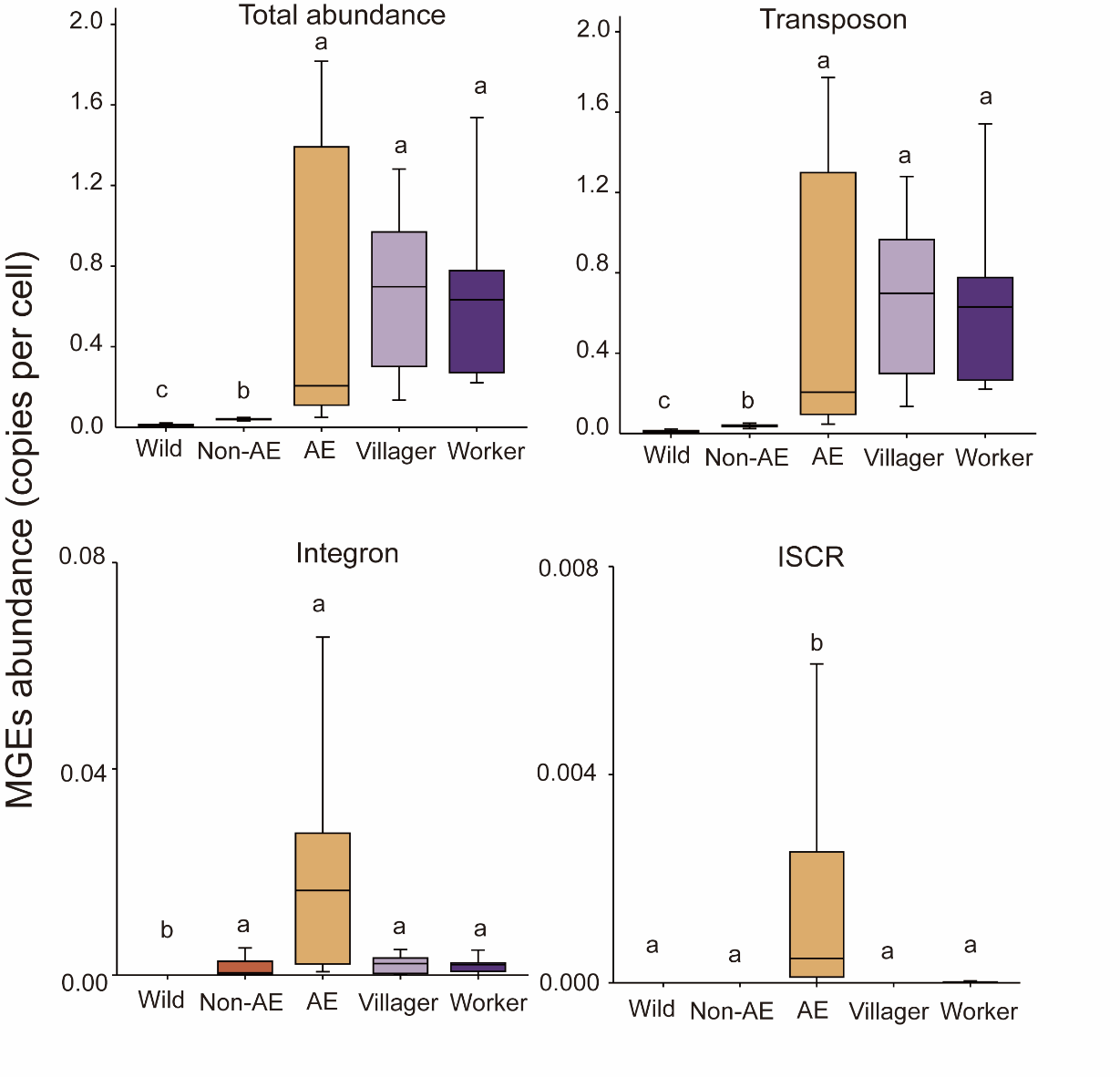


**Fig.****S4** Abundance of mobile genetic elements. Different lowercase letters indicate significant differences between groups (*P* < 0.05, Kruskal-Wallis test). Groups sharing a letter are not significantly different. **AE**: musk deer with recent antibiotic exposure; **Non-AE**: musk deer without antibiotic exposure in the past six months.


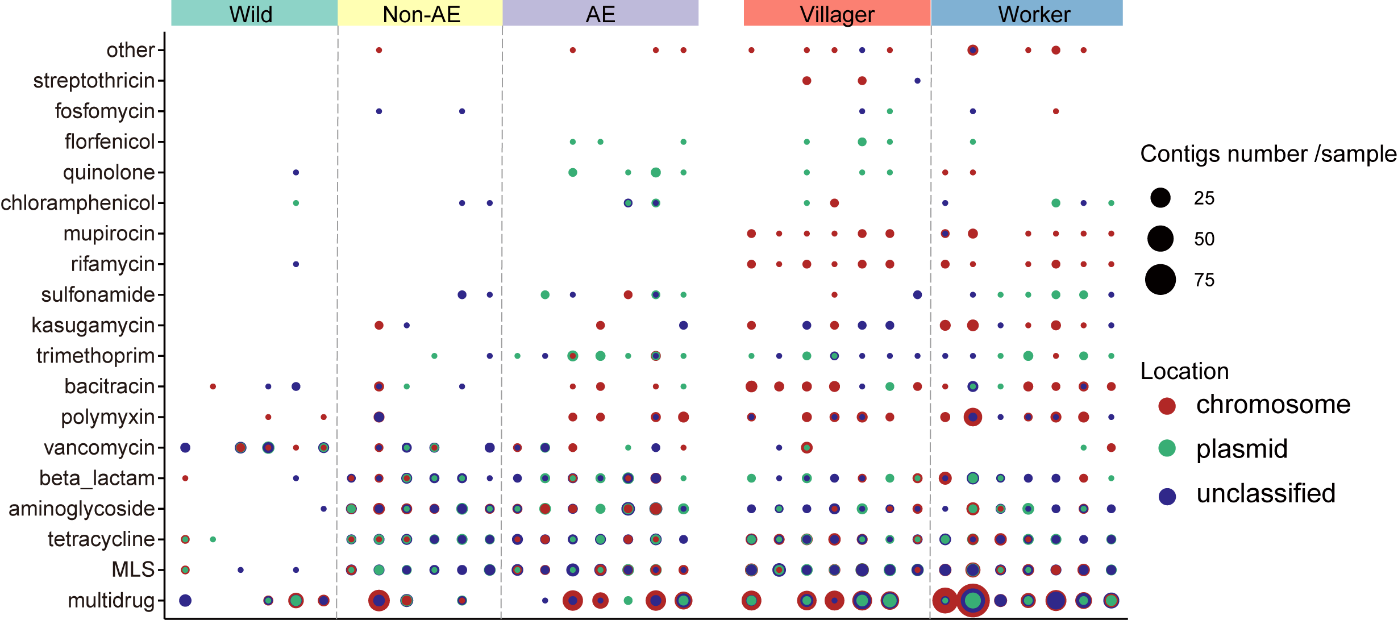


**Fig.S5** Genetic locations of different types of ARGs among musk deer and human fecal samples. **AE**: musk deer with recent antibiotic exposure; **Non-AE**: musk deer without antibiotic exposure in the past six months.


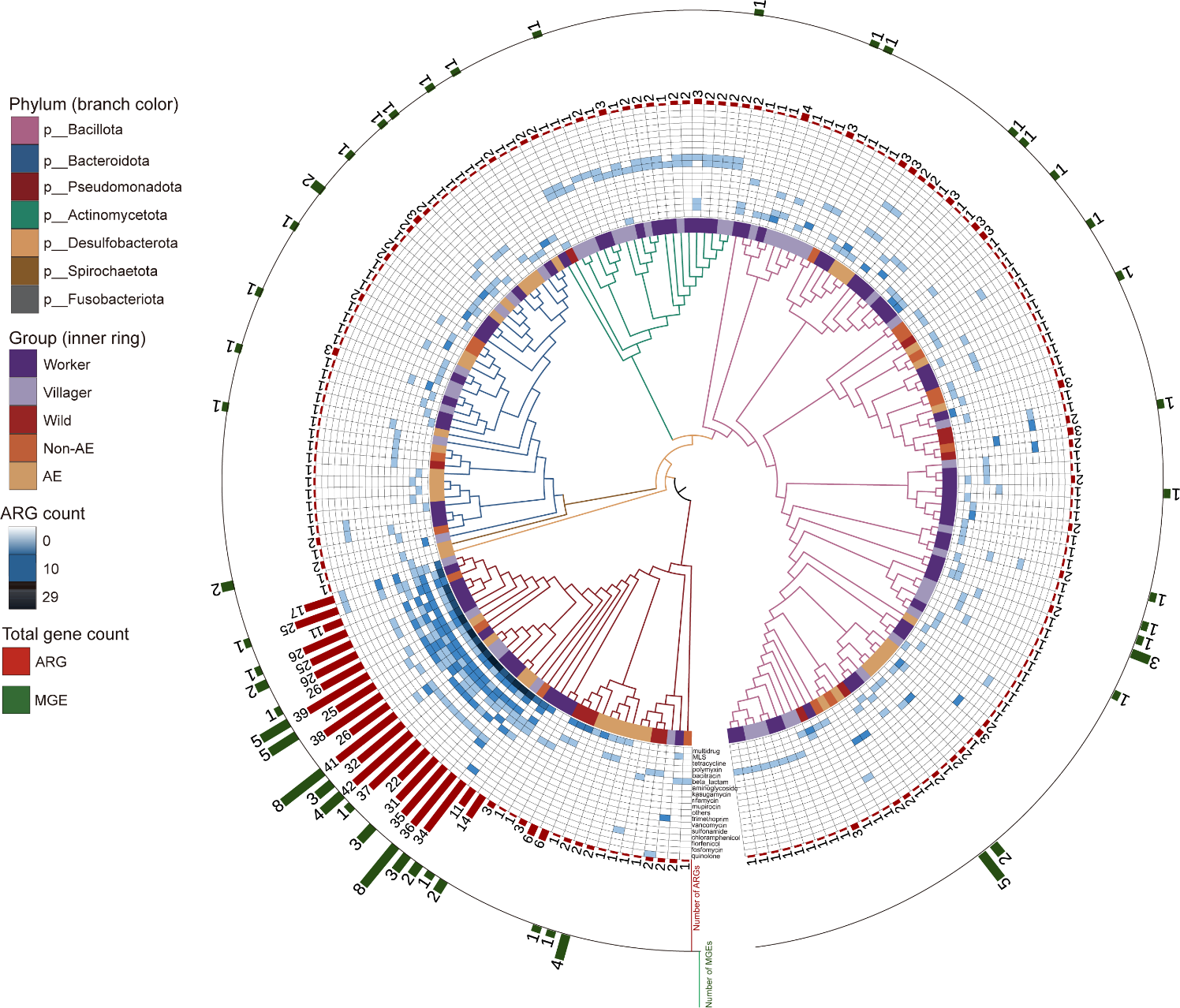


**Fig.S6** Phylogenetic tree of metagenome-assembled genomes (MAGs) reconstructed from musk deer, villagers, and farm worker fecal metagenomes, showing their associated ARGs. Tree branches are colored by bacterial phylum. The most inner ring indicates the sample source groups. The blue heatmap displays the count of ARGs in each MAG, categorized by resistance type. Adjacent bar plots show the total number of ARGs (red) and MGEs (green) detected per MAG. **AE**: musk deer with recent antibiotic exposure; **Non-AE**: musk deer without antibiotic exposure in the past six months.


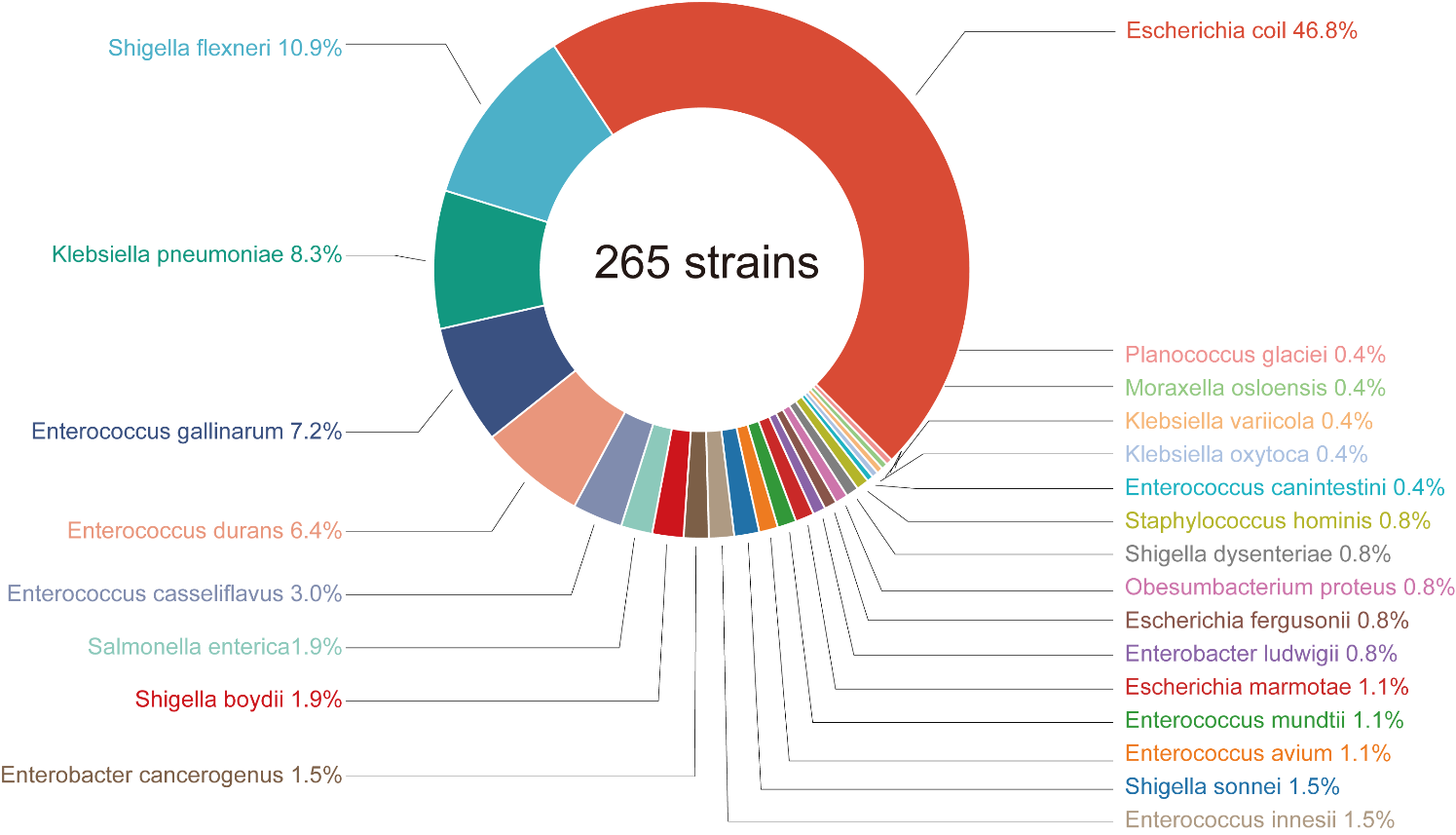


**Fig.S7** Taxonomy of bacterial strains isolated from fecal samples of antibiotic-exposed musk deer.


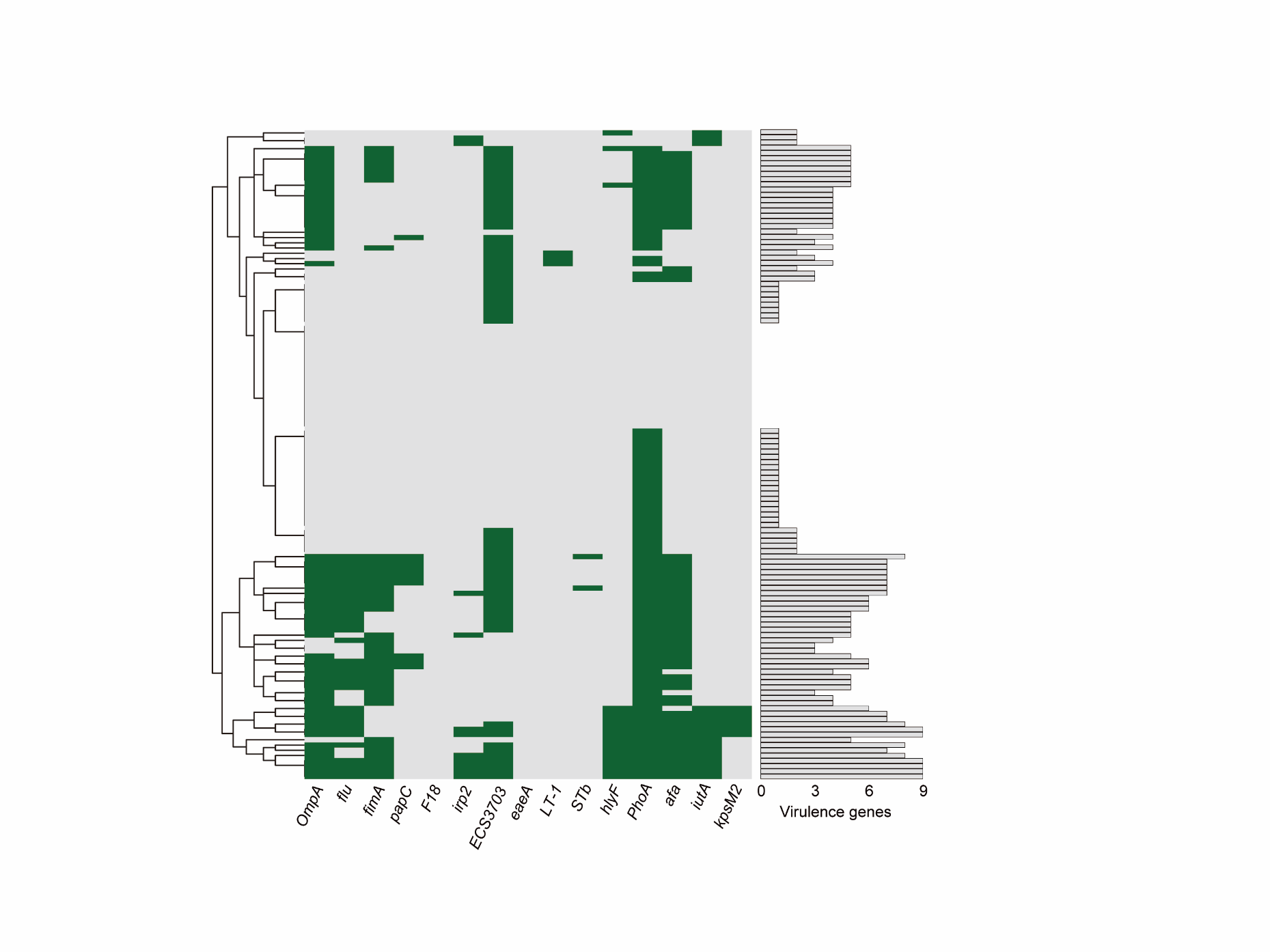


**Fig.S8** Virulence genes detected in *E.coli*. Green boxes indicate the presence of specific virulence factor genes, while grey boxes indicate absence. The bar on the right represents the total number of virulence factor genes identified in each *E.coli* isolate.

**
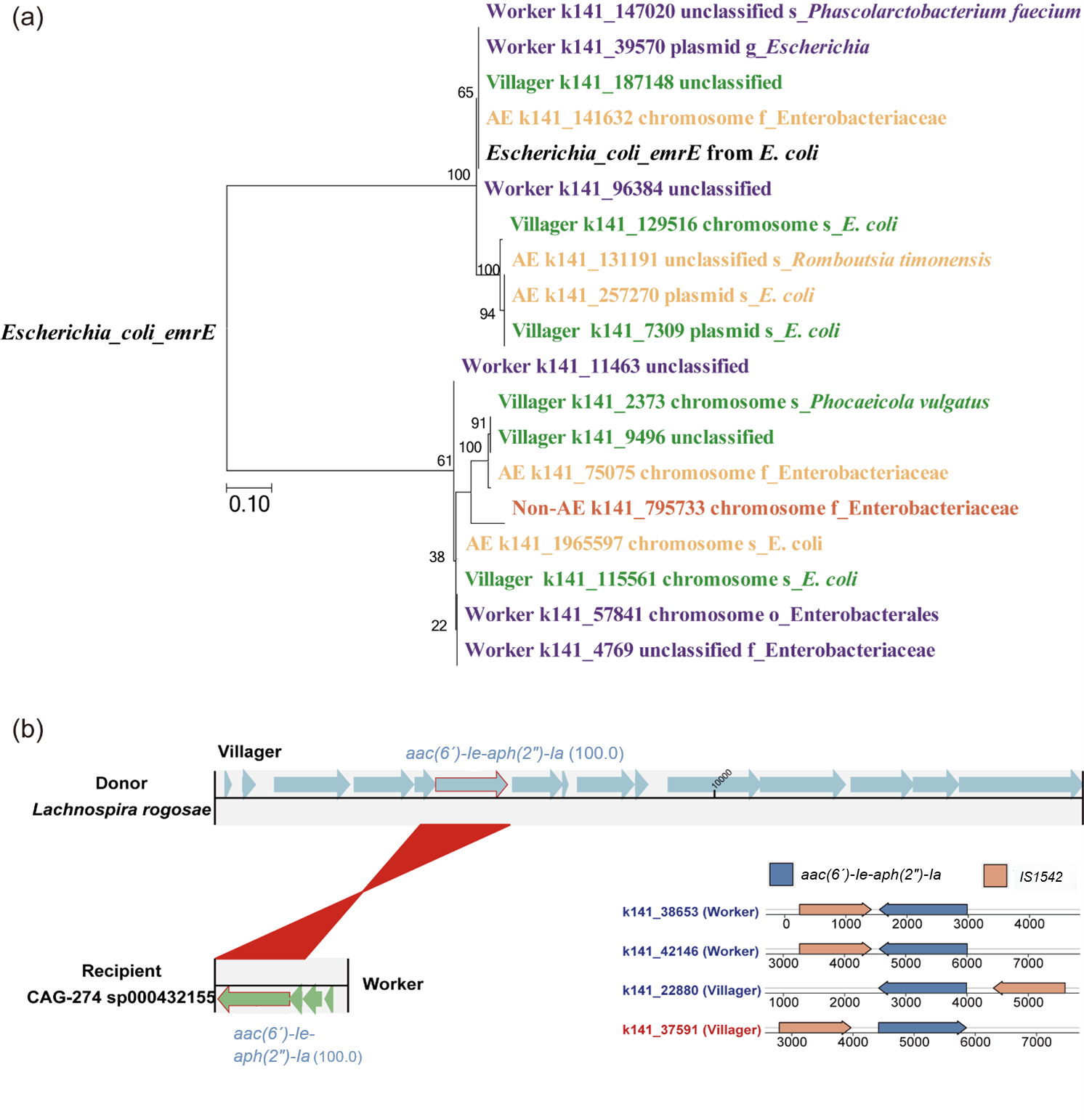
**

**Fig.S9** Transmission of ARGs between captive musk deer and farm workers. (a) Maximum likelihood phylogenetic tree of the *Escherichia_coli_emrE* gene. The italicized black sequence was retrieved from the SARG database. (b) Evidence of horizontal gene transfer of the *aac(6´)-Ie-aph(2")-Ia* gene. **AE**: musk deer with recent antibiotic exposure; **Non-AE**: musk deer without antibiotic exposure in the past six months.

**Supplementary Tables**

**Table S1** Information of metagenomes used in this study.

| **Database** | **Accession number** | **Group** | **Species** | **Sampling location** |
| --- | --- | --- | --- | --- |
| NCBI | SRR24145993 | Farm-captive | *Ovis aries* | / |
| NCBI | SRR24145994 | Farm-captive | *Capra hircus* | / |
| NCBI | SRR24653828 | Farm-captive | *Capra hircus* | / |
| NCBI | SRR24653678 | Farm-captive | *Ovis aries* | / |
| NCBI | SRR24653748 | Farm-captive | *Capra hircus* | / |
| NCBI | SRR24653658 | Farm-captive | *Ovis aries* | / |
| NCBI | SRR24653862 | Farm-captive | *Ovis aries* | / |
| NCBI | SRR24653753 | Farm-captive | *Ovis aries* | / |
| NCBI | SRR24653735 | Farm-captive | *Capra hircus* | / |
| NCBI | SRR24653668 | Farm-captive | *Capra hircus* | / |
| NCBI | SRR24653839 | Farm-captive | *Ovis aries* | / |
| NCBI | SRR24653817 | Farm-captive | *Ovis aries* | / |
| NCBI | SRR24653898 | Farm-captive | *Ovis aries* | / |
| NCBI | SRR24653931 | Farm-captive | *Ovis aries* | / |
| NCBI | SRR24653920 | Farm-captive | *Ovis aries* | / |
| NCBI | SRR24653626 | Farm-captive | *Ovis aries* | / |
| NCBI | SRR24653942 | Farm-captive | *Ovis aries* | / |
| NCBI | SRR24653724 | Farm-captive | *Ovis aries* | / |
| NCBI | SRR24653812 | Farm-captive | *Ovis aries* | / |
| NCBI | SRR24653689 | Farm-captive | *Ovis aries* | / |
| NCBI | SRR24653801 | Farm-captive | *Ovis aries* | / |
| NCBI | SRR24653690 | Farm-captive | *Ovis aries* | / |
| NCBI | SRR24653657 | Farm-captive | *Ovis aries* | / |
| NCBI | ERR13925484 | Farm-captive | *Ovis aries* | / |
| NCBI | ERR13925487 | Farm-captive | *Ovis aries* | / |
| NCBI | ERR13925486 | Farm-captive | *Ovis aries* | / |
| NCBI | ERR13925485 | Farm-captive | *Ovis aries* | / |
| NCBI | ERR13925483 | Farm-captive | *Ovis aries* | / |
| NCBI | ERR4083933 | Wild | *Aepyceros melampus* | / |
| NCBI | ERR4083983 | Wild | *Aepyceros melampus* | / |
| NCBI | ERR4083684 | Wild | *Capra hircus* | / |
| NCBI | ERR4083652 | Wild | *Capra ibex* | / |
| NCBI | ERR4083897 | Wild | *Capra ibex* | / |
| NCBI | ERR4084115 | Wild | *Capra ibex* | / |
| NCBI | ERR4084088 | Wild | *Capra hircus* | / |
| NCBI | ERR4083725 | Wild | *Ovis aries musimon* | / |
| NCBI | ERR4083845 | Wild | *Capra hircus* | / |
| NCBI | ERR4083894 | Wild | *Ovis aries musimon* | / |
| NCBI | ERR4083518 | Wild | *Gazella bennettii* | / |
| NCBI | ERR4083732 | Wild | *Gazella subgutturosa* | / |
| NCBI | ERR4083855 | Wild | *Ovis aries* | / |
| NCBI | ERR4083850 | Wild | *Gazella subgutturosa* | / |
| NCBI | ERR4084110 | Wild | *Capra ibex* | / |
| NCBI | ERR4083656 | Wild | *Ovis aries* | / |
| NCBI | ERR4083687 | Wild | *Capra ibex* | / |
| NCBI | SRR9995037 | Wild | *Gazella subgutturosa* | / |
| NCBI | SRR9995038 | Wild | *Gazella subgutturosa* | / |
| NCBI | ERR4084091 | Wild | *Ovis aries* | / |
| NCBI | SRR9995036 | Wild | *Gazella subgutturosa* | / |
| NCBI | ERR4083755 | Wild | *Rupicapra rupicapra* | / |
| NCBI | ERR4083890 | Wild | *Capra ibex* | / |
| NCBI | ERR4083896 | Wild | *Rupicapra rupicapra* | / |
| NCBI | SRR9995039 | Wild | *Gazella subgutturosa* | / |
| NCBI | ERR4083902 | Wild | *Capra ibex* | / |
| NCBI | ERR4083688 | Wild | *Capra ibex* | / |
| NCBI | SRR24915795 | Zoo-captvie | *Ovis aries* | / |
| NCBI | SRR24915832 | Zoo-captvie | *Capra hircus* | / |
| CNCB | SAMC5241212 | Zoo-captvie | *Naemorhedus goral* | China: Shaanxi |
| CNCB | SAMC5241213 | Zoo-captvie | *Naemorhedus goral* | China: Shaanxi |
| CNCB | SAMC5241211 | Zoo-captvie | *Kobus leche* | China: Shaanxi |
| CNCB | SAMC5241210 | Zoo-captvie | *Kobus leche* | China: Shaanxi |
| CNCB | SAMC5241209 | Zoo-captvie | *Kobus leche* | China: Shaanxi |
| CNCB | SAMC5241216 | Zoo-captvie | *Ovis ammon* | China: Shaanxi |
| CNCB | SAMC5241218 | Zoo-captvie | *Ovis ammon* | China: Shaanxi |
| CNCB | SAMC5241217 | Zoo-captvie | *Ovis ammon* | China: Shaanxi |
| CNCB | SAMC5241214 | Zoo-captvie | *Ovis ammon* | China: Shaanxi |
| CNCB | SAMC5241215 | Zoo-captvie | *Ovis ammon* | China: Shaanxi |
| CNCB | SAMC5241207 | Zoo-captvie | *Capra hircus* | China: Shaanxi |
| CNCB | SAMC5241208 | Zoo-captvie | *Capra hircus* | China: Shaanxi |

**Table S2** The proportion of metagenomic reads mapped to reconstructed MAGs.

| **group** | **mapping rates** | **mapping rates per sample** | **map reads** | **total reads** |
| --- | --- | --- | --- | --- |
| wild | 36% | 38.37% | 36159852 | 94234814 |
|  |  | 69.46% | 60046058 | 86441518 |
|  |  | 29.44% | 19326989 | 65652386 |
|  |  | 30.00% | 19989893 | 66636354 |
|  |  | 17.87% | 11953725 | 66877448 |
|  |  | 33.44% | 22613739 | 67628296 |
| AE | 43% | 45.64% | 35542245 | 77872306 |
|  |  | 37.94% | 25846924 | 68127288 |
|  |  | 51.94% | 35647047 | 68627376 |
|  |  | 22.37% | 16182703 | 72337924 |
|  |  | 40.94% | 28281492 | 69078592 |
|  |  | 39.70% | 26694345 | 67242496 |
|  |  | 62.93% | 57369024 | 91158276 |
| Non-AE | 31% | 43.03% | 31889257 | 74105990 |
|  |  | 37.46% | 27726142 | 74024670 |
|  |  | 28.12% | 20434331 | 72658148 |
|  |  | 27.42% | 18628642 | 67941754 |
|  |  | 27.83% | 20690635 | 74353486 |
|  |  | 21.62% | 14544175 | 67265460 |
| villager | 49% | 46.30% | 34902806 | 75381848 |
|  |  | 63.25% | 57206116 | 90450078 |
|  |  | 52.95% | 37410008 | 70649798 |
|  |  | 47.39% | 36021003 | 76017582 |
|  |  | 39.43% | 28626019 | 72600498 |
|  |  | 35.71% | 24075772 | 67417032 |
|  |  | 57.50% | 44251924 | 76954168 |
| worker | 50% | 42.66% | 28254717 | 66229720 |
|  |  | 39.04% | 25925733 | 66406346 |
|  |  | 44.65% | 36909359 | 82658310 |
|  |  | 40.37% | 27484298 | 68077892 |
|  |  | 53.63% | 35791952 | 66735622 |
|  |  | 64.08% | 47242113 | 73726958 |
|  |  | 68.48% | 51589281 | 75331488 |

**Table S3** PCR primers and conditions used for detecting virulence factors and ARGs.

| **Type** | **Subtype** | **Gene** | **Primer sequence (5’-3’)** | **Product length (bp)** | **Anneal** | **Extend** |
| --- | --- | --- | --- | --- | --- | --- |
| Virulence Factors | Adhesins | *OmpA* | F: ACGCTGTTTCACGTTGTCA | 753 | 54°C 25s | 72°C 45s |
|  |  |  | R: AACCCGTATGTTGGCTTT |  |  |  |
|  |  | *flu* | F: CTGGTATGGAATCACTTACGGG | 965 | 57°C 30s | 72°C 60s |
|  |  |  | R: GAGAATGCTCCCAGGCGGTTTAT |  |  |  |
|  |  | *fimA* | F: GCTCTGGCTGATACTACACC | 495 | 58°C 25s | 72°C 30s |
|  |  |  | R: TTATTGATACTGAACCTTGA |  |  |  |
|  |  | *F18* | F: GTGAAAAGACTAGTGTTTATTTC | 510 | 55°C 25s | 72°C 30s |
|  |  |  | R: CTTGTAAGTAACCGCGTAAGC |  |  |  |
|  |  | *papC* | F: TGATATCACGCAGTCAGTAGC | 501 | 58°C 30s | 72°C 30s |
|  |  |  | R: CCGGCCTATTCACATAA |  |  |  |
|  | pathogenicity island | *irp2* | F: AAGGATTCGCTGTTACCGGA | 301 | 58°C 30s | 72°C 30s |
|  |  |  | R: TCGGCCAGGATGATTCGTCG |  |  |  |
|  |  | *ECS3703* | F: CATGCAATAGTTGCTCAATGC | 552 | 55°C 25s | 72°C 30s |
|  |  |  | R: CCCATTCTCTTTTCGATTCG |  |  |  |
|  |  | *eaeA* | F: CTGAACGGCGATTACGCGAA | 798 | 52°C 30s | 72°C 45s |
|  |  |  | R: CCAGACGATACGATCCAG |  |  |  |
|  | toxins as virulence factors | *LT-1* | F: GGCGACAGATTATACCGTGC | 450 | 54°C 25s | 72°C 45s |
|  |  |  | R: CGGTCTCTATATTCCCTGTT |  |  |  |
|  |  | *STb* | F: GCAATAAGGTTGAGGTGAT | 368 | 60°C 25s | 72°C 25s |
|  |  |  | R: TGTTGTACGAAATCCCCTCTG |  |  |  |
|  |  | *hlyF* | F: TGGCCACAGTCGTTTAGGGTGCTTACC | 450 | 58°C 25s | 72°C 30s |
|  |  |  | R: GGCGGTTTAGGCATTCCGATACTCAG |  |  |  |
|  | others | *PhoA* | F: CGATTCTGGAAATGGCAAAAG | 720 | 55°C 25s | 72°C 45s |
|  |  |  | R: CGTGATCAGCGGTGACTATGAC |  |  |  |
|  |  | *afa* | F: GGCAGAGGGCCGGCAACAGGC | 494 | 61°C 25s | 72°C 45s |
|  |  |  | R: CCCGTAACGCGCCAGCATCTC |  |  |  |
|  |  | *iutA* | F: ATCGGCTGGACATCATGGGAAC | 314 | 61°C 25s | 72°C 45s |
|  |  |  | R: CGCATTTACCGTCGGGAACGG |  |  |  |
|  |  | *KPSM Ⅱ* | F: GCGCATTTGCTGATACTGTTG | 272 | 56°C 30s | 72°C 45s |
|  |  |  | R: CATCCAGACGATAAGCATGAGCA |  |  |  |
| Antibiotic Resistance Genes | beta lactam | *TEM* | F: GTATCCGCTCATGAGACAATA | 717 | 53°C 30s | 72°C 45s |
|  |  |  | R: AGAAGTGGTCCTGCAACTTT |  |  |  |
|  |  | *SHV* | F: ATGCGTTATATTCGCCTGTG | 860 | 56°C 25s | 72°C 50s |
|  |  |  | R: TTAGCGTTGCCAGTGCTCGA |  |  |  |
|  |  | *CTX-M* | F: GGTGTGGTGCGATGAGCACAG | 260 | 55°C 25s | 72°C 30s |
|  |  |  | R: CACGGTTCAGCCATCCCTGAG |  |  |  |
|  | aminoglycoside | *aac(3')-IIa* | F: GGCGACTTCACCGTTTCT | 412 | 54°C 25s | 72°C 30s |
|  |  |  | R: GGACCGATCACCCTACGAG |  |  |  |
|  |  | *aac(6')-Ib* | F: TTGCGATGCTCTATGAGTGGCTA | 482 | 55°C 25s | 72°C 30s |
|  |  |  | R: CTCGAATGCCTGGCGTGTTT |  |  |  |
|  |  | *aacC* | F: ACCCTACGAGGAGACTCTGAATG | 384 | 55°C 25s | 72°C 25s |
|  |  |  | R: CCAAGCATCGGCATCTCATA |  |  |  |
|  | tetracycline | *tetA* | F: GCTACATCCTGCTTGCCTTC | 210 | 58°C 25s | 72°C 20s |
|  |  |  | R: CATAGATCGCCGTGAAGAGG |  |  |  |
|  |  | *tetB* | F: TTGGTTAGGGGCAAGTTTTG | 659 | 58°C 25s | 72°C 40s |
|  |  |  | R: GTAATGGGCCAATAACACCG |  |  |  |
|  |  | *tetC* | F: CTTGAGAGCCTTCAACCCAG | 418 | 58°C 25s | 72°C 30s |
|  |  |  | R: ATGGTCGTCATCTACCTGCC |  |  |  |
|  | quinolone | *gyrA* | F: GGTGACGTAATCGGTAAATA | 810 | 53°C 45s | 72°C 35s |
|  |  |  | R: ACCATGGTGCAATGCCACCA |  |  |  |
|  |  | *qnrB* | F: GATCGTGAAAGCCAGAAAGG | 469 | 53°C 25s | 72°C 30s |
|  |  |  | R: ACGATGCCTGGTAGTTGTCC |  |  |  |
|  |  | *qnrD* | F: TTTTCGCTAACTAACTCGC | 984 | 56°C 25s | 72°C 60s |
|  |  |  | R: GAAAGGATAAACAGGCAAAT |  |  |  |
|  | sulfonamide | *sul1* | F: GTGACGGTGTTCGGCATTCT | 779 | 63°C 30s | 72°C 45s |
|  |  |  | R: TCCGAGAAGGTGATTGCGCT |  |  |  |
|  |  | *sul2* | F: CGGCATCGTCAACATAACCT | 721 | 57°C 25s | 72°C 45s |
|  |  |  | R: TGTGCGGATGAAGTCAGCTC |  |  |  |
|  |  | *sul3* | F: GAGCAAGATTTTTGGAATCG | 880 | 55°C 30s | 72°C 50s |
|  |  |  | R: CATCTGCAGCTAACCTAGGGCTTTGGA |  |  |  |

**Table S4** List of antibiotics used for susceptibility testing.

| **Types of antibiotics** | **Drug Name** | **Abbreviation** | **Dosage per tablet** |
| --- | --- | --- | --- |
| beta-lactam | Cefalexin | CN | 30µg |
|  | Cefazolin | CZ | 30µg |
|  | Cefuroxime Sodium | CXM | 30µg |
|  | Ceftazidime | CAZ | 30µg |
|  | Ceftriaxone Sodium | CTR | 30µg |
|  | Cefoperazone | CPZ | 75µg |
| aminoglycoside | Amikacin | AMK | 30µg |
|  | Gentamicin | GEN | 10µg |
|  | Kanamycin | KAN | 30µg |
|  | Streptomycin | S | 10µg |
| tetracycline | Tetracycline | TET | 30µg |
|  | Minocycline | MI | 30µg |
|  | Doxycycline | DO | 30µg |
| quinolones | Norfloxacin | NOR | 10µg |
|  | Levofloxacin | LEV | 5µg |
| sulfonamide | Cotrimoxazole | SXT | 25µg |
| polypeptide | Polymyxin B | PB | 300IU |
